# The 2023 Impact of Inflammatory Bowel Disease in Canada: Executive Summary

**DOI:** 10.1093/jcag/gwad003

**Published:** 2023-06-01

**Authors:** Joseph W Windsor, M Ellen Kuenzig, Sanjay K Murthy, Alain Bitton, Charles N Bernstein, Jennifer L Jones, Kate Lee, Laura E Targownik, Juan-Nicolás Peña-Sánchez, Noelle Rohatinsky, Sara Ghandeharian, James H B Im, Tal Davis, Jake Weinstein, Quinn Goddard, Julia Gorospe, Eric I Benchimol, Gilaad G Kaplan

**Affiliations:** Departments of Medicine and Community Health Sciences, University of Calgary, Calgary, Alberta, Canada; SickKids Inflammatory Bowel Disease Centre, Division of Gastroenterology, Hepatology and Nutrition, The Hospital for Sick Children, Toronto, Ontario, Canada; Child Health Evaluative Sciences, SickKids Research Institute, The Hospital for Sick Children, Toronto, Ontario, Canada; Department of Medicine, University of Ottawa, Ottawa, Ontario, Canada; The Ottawa Hospital IBD Centre, Ottawa, Ontario, Canada; Division of Gastroenterology and Hepatology, McGill University Health Centre IBD Centre, McGill University, Quebec, Canada; Department of Internal Medicine, Max Rady College of Medicine, Rady Faculty of Health Sciences, University of Manitoba, Winnipeg, Manitoba, Canada; University of Manitoba IBD Clinical and Research Centre, Winnipeg, Manitoba, Canada; Departments of Medicine, Clinical Health, and Epidemiology, Dalhousie University, Halifax, Nova Scotia, Canada; Crohn’s and Colitis Canada, Toronto, Ontario, Canada; Division of Gastroenterology and Hepatology, Mount Sinai Hospital, University of Toronto, Toronto, Ontario, Canada; Department of Community Health and Epidemiology, University of Saskatchewan, Saskatoon, Saskatchewan, Canada; College of Nursing, University of Saskatchewan, Saskatoon, Saskatchewan, Canada; Crohn’s and Colitis Canada, Toronto, Ontario, Canada; SickKids Inflammatory Bowel Disease Centre, Division of Gastroenterology, Hepatology and Nutrition, The Hospital for Sick Children, Toronto, Ontario, Canada; Child Health Evaluative Sciences, SickKids Research Institute, The Hospital for Sick Children, Toronto, Ontario, Canada; SickKids Inflammatory Bowel Disease Centre, Division of Gastroenterology, Hepatology and Nutrition, The Hospital for Sick Children, Toronto, Ontario, Canada; Child Health Evaluative Sciences, SickKids Research Institute, The Hospital for Sick Children, Toronto, Ontario, Canada; SickKids Inflammatory Bowel Disease Centre, Division of Gastroenterology, Hepatology and Nutrition, The Hospital for Sick Children, Toronto, Ontario, Canada; Child Health Evaluative Sciences, SickKids Research Institute, The Hospital for Sick Children, Toronto, Ontario, Canada; Departments of Medicine and Community Health Sciences, University of Calgary, Calgary, Alberta, Canada; Departments of Medicine and Community Health Sciences, University of Calgary, Calgary, Alberta, Canada; SickKids Inflammatory Bowel Disease Centre, Division of Gastroenterology, Hepatology and Nutrition, The Hospital for Sick Children, Toronto, Ontario, Canada; Child Health Evaluative Sciences, SickKids Research Institute, The Hospital for Sick Children, Toronto, Ontario, Canada; ICES, Toronto, Ontario, Canada; Department of Paediatrics, Temerty Faculty of Medicine, University of Toronto, Toronto, Ontario, Canada; Institute of Health Policy, Management and Evaluation, Dalla Lana School of Public Health, University of Toronto, Toronto, Ontario, Canada; Departments of Medicine and Community Health Sciences, University of Calgary, Calgary, Alberta, Canada

**Keywords:** Comorbidities, Crohn’s disease, Issues affecting the IBD community, Treatment, Ulcerative colitis

## Abstract

The burden of inflammatory bowel disease (IBD) (i.e., associated direct and indirect costs, prevalence of disease, personal impact to the individual and to caregivers) continues to increase in Canada. The prevalence of IBD has increased since Crohn’s and Colitis Canada’s 2018 Impact of IBD report from an estimated 270,000 Canadians living with IBD in 2018 to an estimated 322,600 Canadians living with IBD today in 2023. Consequently, associated costs of IBD have also dramatically increased from an estimated $2.57 billion in 2018 to an estimated $5.38 billion in 2023; this increase is due to multiple factors including increased prevalence of disease, inflation, and additional identified factors (e.g., presenteeism, costs of childcare). Beyond the economic impact of IBD, these diseases have a significant impact on people living with the disease and their caregivers, including different presentations of disease, different commonly associated extra-intestinal manifestations or comorbid conditions, and different barriers to accessing care. In this supplementary issue, we review: Evolving trends in the epidemiology of IBD; updated estimates of indirect and direct costs (including out-of-pocket costs) associated with IBD; information specific to IBD in children, adolescents, and seniors; issues related to IBD pertaining to sex and gender; information specific to risks associated with COVID-19 and cancer related to IBD; an overview of current treatments for IBD; and evolving care models, including access to care.

**Key Points**

1. The epidemiologic trends of IBD in Canada show similar patterns to those reported in Crohn’s and Colitis Canada’s 2018 Impact of IBD report. The prevalence of disease is continuing to grow rapidly such that approximately 470,000 (1.1% or 1 in 91) Canadians are forecast to be living with IBD by 2035.
2. IBD imparts a significant cost to individuals with the disease, family caregivers of those individuals, the economy, and healthcare systems. In Canada, in 2023, IBD is estimated to cost $5.38 billion in combined direct, indirect, and out-of-pocket costs ($2.05 billion in indirect and out-of-pocket, $3.33 billion in direct), and this may be an underestimate of the true burden.
3. The impact of IBD on specific populations (e.g., children and adolescents, seniors, Indigenous peoples, immigrants to Canada, members of the LGBTQ2S+ community, or those of disadvantaged socio-economic status) is heterogeneous; these subpopulations experience different complications of IBD and/or different barriers to accessing care.
4. Several medical therapies used to treat IBD suppress the immune system of those being treated. However, only high-dose corticosteroids (>20 mg/day) was found to increase the risk of severe COVID-19 (e.g., hospitalization, ICU admission, death). Continuing medical therapy to maintain disease remission and receiving booster vaccines against SARS-CoV-2 are important factors in mitigating COVID-19 risks.
5. Colorectal cancer occurs 1.5 to 2 times more often in people with IBD compared to the general population, although the absolute risk of cancer remains low. Regular, high-quality surveillance colonoscopy is important for early detection and management of neoplastic lesions.
6. Psychiatric disorders are 1.5 to 2 times more prevalent in people with IBD compared to the general population, and nearly one quarter to one third of people with IBD experience elevated symptoms of depression or anxiety. Clinical guidelines recommend screening those with IBD for mental health concerns and recognize the need for clinical care pathways when these concerns are detected.

## INTRODUCTION

In 2023, more than 320,000 Canadians are estimated to be living with inflammatory bowel disease (IBD). IBD is a chronic disease characterized by periods of relapsing and remitting inflammation of the digestive tract. It comprises Crohn’s disease and ulcerative colitis, though some individuals may be diagnosed with IBD-unspecified. Chronic inflammation and the medications used to treat IBD may increase the cumulative risk of some cancers over the general population, though the absolute risk of cancer remains low. Further, because some medications used to treat IBD suppress the immune system, there can be an increased risk of infections, such as SARS-CoV-2 leading to COVID-19.

IBD affects portions of our population in different ways: Children and adolescents who are diagnosed with IBD will live with long-standing disease since they are unlikely to die due to complications of the disease; seniors who are diagnosed with IBD will face issues around age-related co-morbidities and polypharmacy; differences in sex and gender are correlated with different healthcare utilization patterns and needs; and different ethnic groups, such as Indigenous peoples or immigrant communities in Canada, experience additional barriers to healthcare.

IBD imparts a significant burden on Canadian healthcare systems in terms of direct costs and resources; the employment sector in terms of lost productivity; caregivers to individuals living with the disease, such as family members who are faced with indirect and out-of-pocket costs related to assisting the individual in accessing healthcare; and those living with the disease, who may experience IBD-related health concerns and out-of-pocket costs for accessing care.

Crohn’s and Colitis Canada’s 2018 IBD Impact report summarized the burden of IBD in Canada at that time (1). This new 2023 report updates the Canadian literature over the five past years and expands the topics of the previous report to present updated information; discuss what the future of care may be; and identify knowledge gaps, future research areas, and key policy and advocacy outcomes.

## METHODS

The Steering Committee of the 2023 Impact of IBD in Canada report met in 2022 to identify one to three co-leads for each of the articles comprising the 11 topics of the report: Epidemiology, Indirect (Individual and Societal) & Direct Out-of-Pocket Costs, Direct Health System & Medication Costs, Children & Adolescents, Seniors, Sex & Gender, Mental Health, COVID-19, Cancer, Treatment Landscape, and Access to Care. Each of the co-leads identified a working group to research, write, and review the articles. The Steering Committee, along with the various co-leads, made final revisions to the articles. Research informing each of the articles was taken from the working groups’ specialist knowledge and supported by a systematic literature review. Key points, knowledge gaps and future research directions, and key advocacy points, along with a paragraph entitled, Patient & Caregiver Perspective, were informed through engagement of patient and caregiver partners.

### Systematic Review

We conducted systematic literature searches to inform the following chapters of the impact report: Access to Care, Cancer, Children & Adolescents, COVID-19, Direct Health System & Medication Costs, Indirect (Individual and Societal) & Direct Out-of-Pocket Costs, Epidemiology, Mental Health, Seniors, Sex & Gender, and Treatment Landscape. Of these, four chapters (Children & Adolescents, Direct Health System & Medication Costs, Seniors, and Treatment Landscape) included multiple systematic reviews to address specific sections within each chapter. Where there was overlap between sub-topics within chapters, the relevant studies from one review were included in additional reviews. Information for the Epidemiology chapter was based on two systematic reviews, one on the epidemiology of pediatric IBD conducted by Kuenzig et al. (2), and one on the epidemiology of adult IBD conducted by Windsor et al. in 2022 (3). The specific databases searched varied by the topic of each search. MEDLINE (Ovid) and EMBASE (Ovid) were searched for all systematic reviews. Topic-specific searches also included PsycINFO (Ovid), CINAHL (EBSCO), Cochrane Library (Wiley), and/or medRxiv. All searches were conducted between April and June 2022 and included studies published in 2018 or later, in order to update the 2018 Impact of IBD in Canada report. The COVID-19 literature search included studies published in 2021 or later, which updated the 2021 Impact of COVID-19 and IBD in Canada report. These dates were chosen to minimize overlap in identified studies with previous iterations of this impact report (1,4). Review topics, search terms, databases, and the exact date of the literature search for each review are specified in Supplementary Material 1.

We included any study addressing the specified topic(s) of each review. We included primary studies, clinical practice guidelines, and recommendations. We excluded conference abstracts, studies that were not published in English, study protocols, case series of <5 individuals with IBD, and basic or translational science studies. Editorials, comments, and letters were excluded if they did not include original data. Systematic reviews were included in all reviews. At the specification of chapter authors, narrative reviews were included in some chapters but not others. Chapter-specific inclusion and exclusion criteria are outlined in Supplementary Material 1.

InsightScope was used to facilitate the abstract and full-text review process. Abstracts and full texts of studies identified from the literature search were independently screened by two individuals for each chapter. Conflicts at both stages were reviewed by a third reviewer (M.E.K.). Study objective and design were extracted for each study by at least two reviewers. Studies pertinent to each chapter of the report were then distributed to authors of the relevant chapters. Included studies were synthesized qualitatively. Additional sources were incorporated based on each working group’s areas of expertise.

### Patient and Caregiver Partner Engagement

Crohn’s and Colitis Canada invited individuals, via email, who had past engagement with the organization to assist in the development of the 2023 Impact of Inflammatory Bowel Disease in Canada report. Individuals were included if they were living with or caring for someone with Crohn’s disease or ulcerative colitis. The purpose of this partnership was to gather perspectives of patients and caregivers for each article including information on: (i) content most relevant and meaningful to them as persons affected by IBD, (ii) key findings or take-away messages, (iii) knowledge gaps, and (iv) advocacy priorities for Crohn’s and Colitis Canada. Each article within this report contains a Patient & Caregiver Perspective paragraph comprising the key feedback from the partners reviewing each respective article. This paragraph is in the partners’ words and is not edited by the working group of the article or the Steering Committee of the report.

Fifty-four patient and caregiver partners were recruited, and 46 provided their voluntary consent via SurveyMonkey (see Supplementary Material 2). Of these partners, the majority (38) were living with IBD (53% with Crohn’s disease, 21% with ulcerative colitis, 26% did not specify IBD type) and 17% (9) were caregivers to children living with IBD. One patient partner was both living with IBD and a caregiver to a child with IBD.

Partners were located across Canada: 41% (19) residing in Ontario, 13% (6) in Alberta, 9% (4) in Quebec, 7% (3) in Saskatchewan, 7% (3) in British Columbia, 7% (3) in Newfoundland, 4% (s) in Nova Scotia, 4% (2) in Prince Edward Island, 4% (2) in Manitoba, and 2% (1) in New Brunswick. Four partners were Indigenous, from communities within Saskatchewan and Manitoba.

Partners were from across the lifespan, including 36% (17) that were 18 to 29 years of age, 42% (19) were 30 to 44 years of age, 4% (2) were 45 to 59 years of age, 4% (2) were 60 to 64 years of age, 7% (3) that were 70+ years of age, and 7% (3) did not specify age. The majority (72%) of partners self-identified as being female gender, 24% (11) self-identified as male, 2% (1) self-identified as transgender, and 2% (1) did not specify gender. For most partners (72%), their highest-level education was a post-secondary degree, 4% completed their high school diploma, and 22% did not specify their highest-level of education.

### Consensus Meeting

After completion of the draft of each chapter and the patient and caregiver partner engagement, the Steering Committee, working group co-leads, and partner representatives participated in a two-day virtual consensus meeting. During the consensus meeting, the content of the report was reviewed; partner engagement feedback was incorporated; and the wording of the key points, knowledge gaps and future research directions, and key policy and advocacy points were finalized. Drafts were then finalized by each working group and underwent professional editing by the editor of the report. A blind peer review process was facilitated by the *Journal of the Canadian Association of Gastroenterology* and working group leads incorporated anonymous feedback from the peer reviewers to strengthen the chapters.

## Report Summary

### Epidemiology

Although there are differences in the epidemiology of IBD across Canadian regions (e.g., between provinces, between rural versus urban areas), Canada is in the third epidemiologic stage of IBD evolution where the rate of increasing incidence slows, stabilizes, or even declines, but the prevalence of disease rapidly increases—this stage of IBD evolution is known as Compounding Prevalence (5). Today, it is estimated that approximately 322,600 Canadians are living with IBD, which is approximately 0.82% of our population; this total increases every year with new cases of pediatric-onset IBD being the primary driver since the incidence of pediatric-onset IBD is increasing at an average of 1.23% year over year but the incidence of adult- or elderly-onset IBD is stable. If the historical trends in the incidence and prevalence of IBD continue, it is estimated that approximately 470,000 Canadians will be living with IBD by 2035, which is approximately 1.1% of our population, or 1 in every 91 people in the country.

IBD was once considered to be a disease of Caucasian people of European descent. Today it is recognized among all populations and is found on every populated continent. It is also diagnosed in immigrants and the children of immigrants to Canada, and among Indigenous peoples in Canada. Several current research projects such as the Crohn’s and Colitis Canada Genetic, Environmental, and Microbial project (CCC-GEM) are focused on discovering factors that influence the development of IBD; by understanding these factors, this research offers possible strategies surrounding diet, lifestyle, behavioural, and environmental modifications to improve the healthcare and quality of life for those with IBD.

### Indirect (Individual and Societal) and Direct Out-of-Pocket Costs

The burden of IBD affects the individual, those who care for the individual, and society through direct, out-of-pocket costs for things such as medical supplies not covered by insurance (e.g., ostomy supplies, medications for bowel preparation prior to colonoscopy, dispensing fees); allied healthcare; and additional costs pertaining to diet, vitamins, and supplements. Additionally, IBD imparts indirect costs on the individual, those who care for them, and society in the form of lost productivity, premature retirement or death, and an impact on the maximum potential of the individual.

Medical supplies and other patient-borne costs associated with seeking care not covered by insurance account for approximately $536 million in out-of-pocket costs for Canadians, annually. The indirect costs of IBD can be estimated from costs associated with lost opportunity: Delayed entry into the workforce, premature retirement or death, and impacts on maximum earning potential. Further, lost opportunity also refers to other, intangible costs of IBD that cannot be measured such as the impact on quality of life. The indirect costs of IBD in Canada are estimated at $1.51 billion, annually, but this is likely an underestimate. Another type of indirect cost associated with IBD is that incurred by society or the employer; in addition to the reduced earning potential the individual suffers due to lost opportunity, society also bears the cost of things like unemployment or early retirement. Unemployment is the largest contributor to indirect costs of IBD in Canada, estimated to account for $1.14 billion, annually. Even while an individual with IBD is working, employers may bear indirect costs associated with lost productivity through absenteeism (i.e., missed work hours) and presenteeism (i.e., reduced productivity while at work). The costs of absenteeism and presenteeism are estimated at $285 million, annually.

Finally, the costs to caregivers for those with IBD must also be highlighted. People caring for individuals with IBD must also sacrifice time in helping afflicted individuals seek care such as providing transport to clinics or hospitals for infusions or colonoscopies; this potentially increases the burden in terms of absenteeism, for example.

### Direct Health System and Medication Costs

The direct costs of IBD to health systems in Canada stem from healthcare utilization and medication costs. Healthcare utilization accounts for costs associated with visits to the emergency department (ED), hospitalizations, surgeries, surveillance, and outpatient visits. Combined with the estimated costs of medications, these costs are somewhere between $2.19 and 4.47 billion, annually. Although biologic medications made up roughly 50% of these costs in 2017 and likely account for an even greater percentage of healthcare spending today, there has been an overall reduction in costly health services utilization (i.e., surgeries and hospitalizations); however, there is heterogeneity in this metric based on different sub-populations.

The overall frequency of children presenting to the ED has increased, but the overall frequency of adults and seniors presenting to the ED has decreased; however, due to an increasing number of Canadians living with IBD, the absolute numbers have increased. Disparities in health services utilization varies by province, but there are also within-province differences such that Indigenous people are more likely than the general IBD population to be hospitalized; children with Crohn’s disease of South Asian descent are more likely to be hospitalized at diagnosis, but subsequently have a similar hospitalization rate to the general IBD population; immigrants to Canada who are later diagnosed with IBD have greater health services utilization, but a lower risk of surgery than the general IBD population; and people with IBD living in rural areas are more likely to visit the ED or be hospitalized than those living in urban centres, but there is no statistical difference in the risk of surgery between the two populations; and those of lower socioeconomic status living with IBD are more likely to be hospitalized, undergo surgery, or require systemic corticosteroids.

### Special Populations—Children and Adolescents with IBD

Increasing incidence of IBD in Canada is primarily driven by pediatric-onset disease, and the number of new cases in this population is quickly rising, especially among those under six years old. The presentation of IBD in children and adolescents is different from adult- or elderly-onset disease and comes with a unique set of complications, including the potential for growth stunting, delay of puberty, and deficits in bone development, let alone the mental health costs of having a chronic disease at a young age. Children and adolescents developing IBD frequently have more extensive disease, although those with very early onset IBD (VEO-IBD, presenting under 6 years of age) are more likely to have colonic disease only.

The unique presentation of disease among children and adolescents comes with unique healthcare needs: The treatment options for pediatrics are limited, partially due to children not typically being included in the initial clinical trials for new biologics and small molecule medications, and thus fewer medications being approved for use in pediatrics. In addition, physicians avoid corticosteroids in this population in favour of dietary therapy or biologics because of the possible side effects that steroid use can elicit, such as exacerbating linear growth delay, issues of bone health/development, or exacerbating mental illness. Additionally, roughly 3% of children who develop IBD have a monogenic form of the disease that often presents with other comorbid conditions, immunodeficiencies, or extra-intestinal manifestations (6). Finally, children with IBD display higher rates of anxiety and depression than other children, and also compared with adults with IBD. The transition between pediatric to adult care is a time when psychosocial and physical development is at a critical timepoint—both research into understanding the risks at this timepoint as well as clinical guidelines to successfully transition the care of these individuals are needed.

### Special Populations—Seniors with IBD

By 2035, it is estimated that 1 in 91 Canadians will have IBD; in 2023, that number is already 1 in 88 seniors, up from 1 in 160 in 2018, and the prevalence of IBD in this population continues to increase due to an aging IBD population and new diagnoses among seniors. Seniors with IBD have unique healthcare challenges due to long standing disease (for those diagnosed earlier in life), frailty, and polypharmacy from treatment of comorbid conditions related to aging. The complex healthcare needs of this population are putting additional strain on IBD clinics to provide adequate care; gastroenterologists treating IBD in seniors need to be part of a multidisciplinary team that can avoid adverse effects of polypharmacy and adequately care for longstanding IBD in addition to the sequalae of aging.

Several treatment options for managing IBD in seniors exist but may be limited by medical therapies that have lost effectiveness over a longstanding disease course (for those who were diagnosed earlier in life), or by potential side effects. For example, anti-TNF medications may increase the risk of infections, such as pneumonia, but newer biologics or IL-12/23 inhibitors have better safety profiles regarding infection risk. However, vaccines against preventable infections (e.g., pneumococcus, SARS-CoV-2, herpes zoster) are safe and strongly encouraged to protect seniors with IBD.

In addition to complex healthcare needs, seniors with IBD also face unique barriers to care due to potential mobility restrictions, logistic challenges, or a lack of a support system to advocate on their behalf. While the COVID-19 pandemic rapidly evolved virtual and remote care options that increase the ability to access care for seniors, resources for technological literacy and support are still necessary to prevent modern access options from becoming an additional barrier to care.

### The Influence of Sex & Gender on Canadians Living with IBD

Overall, IBD is not considered a disease that is differentiated in terms of sex. However, sex-specific physiologic states such as puberty, menstruation, pregnancy, or andropause/menopause can have effects on an individual’s IBD, and IBD can, in turn, impact those states. For example, people with IBD who become pregnant are more likely to experience adverse outcomes, including pregnancy loss, low birth weight, preterm birth, or the need for caesarean delivery. IBD is also a stated reason for some females choosing to remain childless, which may be influenced by uncertainty around safety, lack of disease-specific/medication-specific IBD knowledge around pregnancy, or lack of access to pregnancy in IBD specialists.

Similarly, gender in and of itself is not thought to impact the disease course of IBD, but ascribed gender roles—as correlated with sex-specific healthcare utilization patterns—do show different trends among men and women. For example, women are more likely to seek healthcare than men, but are also more likely to receive fragmented care or have IBD symptoms confused with female-specific conditions such as menstrual cramping or endometriosis. Because much of the research available on gender differences in healthcare utilization and trends in the care of IBD rely on administrative healthcare databases that characterize included individuals based on sex, there is very little information on healthcare trends among individuals who do not identify as male or female, transgender individuals, or individuals from the queer community, who may also experience barriers to healthcare or be reluctant to seek care (7).

Although research exists on corollaries between sex- or gender-specific healthcare in IBD, such as an association between oral contraceptive use among females and subsequent IBD diagnosis or trends in seeking healthcare based in men versus women, there is a paucity of research into direct questions of the influence of sex or gender on IBD diagnosis, treatment, or access to care. Due to a large IBD community with highly engaged individuals along with access to longitudinal healthcare databases, Canadian researchers are well positioned to make advances in this important and necessary area.

### Mental Health and IBD

Diagnoses of psychiatric disorders, such as depression and anxiety, are 1.5 to 2 times more common among those with IBD than the general Canadian population, with a greater prevalence among women and people with Crohn’s disease. Children with IBD are at an additional two times the risk for a psychiatric diagnosis and six times the risk for depression. In addition to the challenges that mental illness imparts on an individual, depression and anxiety are known to negatively impact the disease course of IBD, which is a bidirectional phenomenon where active IBD symptoms can also negatively impact mental health conditions.

Psychological treatments for anxiety and depression have been shown to successfully reduce symptoms and significantly improve the quality of life for those with IBD, including helping to mitigate the effect that active symptoms from these mental conditions have on IBD activity. Further, a promising area of intervention is on building and strengthening resilience. The evidence surrounding antidepressant therapies for mental health or IBD outcomes is less robust. In adolescents transitioning from pediatric to adult care, strengthening the individual’s self-autonomy with regards to disease self-management can ease the transition process and may help prevent negative outcomes for the individual.

Due to the association between mental health concerns and IBD, clinical guidelines now recommend both screening for mental health concerns as part of IBD care as well as having an established clinical care pathway when mental health concerns are detected. As it is recommended that gastroenterologists screen for mental health issues, it may be that many gastroenterologists are not equipped to deal with active psychiatric symptoms. Hence, it is further recommended that gastroenterologists be part of a multidisciplinary IBD care team that should include specialists in psychological care.

### COVID-19 and IBD

On March 11, 2020, the World Health Organization declared SARS-CoV-2 a global pandemic; on March 12, 2020, Crohn’s and Colitis Canada formed the COVID-19 and IBD Taskforce to assess, synthesize, and communicate relevant medical and public health information to the IBD community through a dedicated website (with more than 800,000 visits as of Fall 2022) and a series of 30 community-oriented webinars (viewed over 81,000 times as of Fall 2022). The expertise gained from this intense knowledge translation strategy provides a proven strategy for communication in any future public health emergencies.

Three years into the COVID-19 pandemic, there is robust evidence to suggest that most clinical therapies used to treat IBD do not put those with IBD at greater risk of acquiring COVID-19, or experiencing severe COVID-19, compared to the general population. The exception is that people under 50 years of age experiencing a severe IBD flare, or those who require >20 mg per day of corticosteroids are at increased risk of severe COVID-19. Severe outcomes of COVID-19 are defined as hospitalization, ICU admission, or death. Further, despite the fact that people with IBD were not included in clinical trials for COVID-19 vaccine safety and efficacy, there is now robust data to show that these vaccines are safe and elicit excellent immune responses in those with IBD; however, there is a blunted antibody response for those taking prednisone and those on anti-TNF therapies.

As variants of the SARS-CoV-2 virus continue to circulate in the population and breakthrough infections occur in those who have been previously vaccinated, it is strongly recommended that those with IBD continue to receive booster doses of the vaccine, especially now that bivalent vaccines are available that specifically target Omicron subvariants of the virus.

### Cancer and IBD

IBD increases the risk of colorectal cancer by 1.5 to 2 times that of the general population, exacerbated by age, duration of disease, extent and severity of colorectal involvement, and family history of colorectal cancer. However, the absolute risk of colorectal cancer does remain low. In addition to effective treatment of inflammation, regular colonoscopy surveillance is the cornerstone of colorectal cancer prevention in IBD. Some extra-intestinal cancers are also more prevalent in those with IBD, including melanoma, hepatobiliary cancer (especially in those with comorbid primary sclerosing cholangitis), and lung cancer. Having an extra-intestinal cancer may impact the available options to treat IBD.

Additionally, some immunosuppressive therapies commonly used to treat IBD are associated with increased risks of certain extra-intestinal cancers (i.e., increased risks of lymphoma and non-melanoma skin cancers with thiopurine treatment). However, the absolute risks of these cancers remain exceedingly low and should not be a deterrent to effective treatment, particularly since untreated active inflammation increases the risks of disease-related complications and intestinal cancers. Active surveillance is recommended for early detection of neoplastic lesions of the skin, cervix, and hepatobiliary system.

Individuals with IBD are living longer due to advances in IBD treatments and general societal health. As such, the cumulative life-long risk of cancer is increasing in this population. As practitioners face increasing cancer burden in IBD populations, treatment decisions will become more complex and will need to factor in potential risks of cancer.

### Treatment Landscape

Over the past two decades, several biologic and small molecule advanced therapies have become available to treat IBD. These medications have been shown to be safe and effective in clinical and real-world studies and have radically changed the prospect of long-term disease control for those with IBD. Biosimilar therapies have started to emerge for several classes of biologic medications, bending the cost curve downwards through competitive pricing and increasing access to biologic treatments. Evolving treatment paradigms, including treating to a target of objective disease remission (as opposed to resolving symptoms alone) and introducing combination therapies earlier in the disease course have the potential to further improve long-term prognosis. Being able to identify the right treatment for the right individual at the right time in their disease course to optimize long-term prognosis is an important future goal in IBD management and an active area of IBD research.

Although medical management of IBD has substantially advanced, surgery continues to play an important role in those with stricturing, penetrating, or perianal fistulizing Crohn’s disease, medically refractory disease, and colorectal cancer.

Beyond medication and surgical interventions, evidence for alternative therapies that are directed at modifying intestinal bacteria through diet modification, probiotics, and fecal transplants have all shown promise in preliminary studies; however, further research in these areas is needed before there can be widespread recommendation for these alternative or supplemental therapies.

### Access to and Models of Care

The quality of care varies across Canada by geographic differences (e.g., province/territory or urban versus rural living), socio-economic status, and ethnicity (e.g., immigrant or Indigenous communities). Some of the barriers to accessing quality healthcare identified by individuals living with IBD include long wait times for specialist or non-specialist care, the lack of resources surrounding travel times to clinics, and inadequate availability of specialist resources. The latter barrier includes difficulties with access to gastroenterologists, non-invasive surveillance options (e.g., intestinal ultrasound), and allied healthcare professionals to provide help for mental health or other concerns that are frequent among those with IBD. Increasing access to these patient-identified healthcare resources can improve patient-reported outcomes and the quality of life for those living with IBD.

Gaps in accessing care and treatment for IBD, including gaps in psychosocial care, could be addressed partly by implementing standardized quality of care indicators and clinical pathways, such as those proposed by Crohn’s and Colitis Canada’s Promoting Access Centres of Excellence (PACE), with a focus on integrated care management by a multidisciplinary care team.

Access to care, not only for those living in rural areas or who have mobility restrictions but for all individuals with IBD, may be enhanced through the expansion and adoption of virtual health platforms. These platforms could improve communication between individuals living with IBD and their healthcare providers, increase the ability of physicians to implement evidence-based care based on individual data monitoring, and suggest surveillance approaches with the individual’s quality of life and ability to engage in shared decision-making as a focus.

## Recommendations

- The incidence and prevalence of IBD in Canada needs to be continually monitored and updated to constantly understand the overall burden and the change of burden in Canada. These trends should be updated and used as a basis for recommendations to the healthcare system to adjust for the impending burden.
- Canadian healthcare should evolve to include multidisciplinary care, including access to mental health professionals, dietitians, and other allied healthcare professionals to improve the quality of life for people living with IBD. Additionally, extended health benefit providers should be lobbied to provide additional benefits regarding these costs to reduce overall health expenditures.
- The number of people living with IBD in Canada is increasing. At the same time, the costs of treating each person living with IBD are also increasing. The increasing costs of caring for a rising number of people living with IBD are not sustainable indefinitely. Crohn’s and Colitis Canada should advocate for better regulation of the cost of biologic therapies to ensure the financial viability of providing the right medication to the right person at the right time. Both government and private health insurance plans should provide financial pressure on the pharmaceutical industry to lower medication costs.
- Rates of pediatric IBD are rising in Canada. These individuals require multi-disciplinary and specialized care for their chronic disease. They should have access to expert physicians, nurses, dietitians, social workers, pharmacists, and mental health specialists to treat both the individual and their family, no matter where in Canada they live.
- Ensuring that seniors with IBD have access to gastroenterology care is essential; remote access is also important because there may be some barriers in some forms of virtual care. Tele-medicine should be accessible and be funded to ensure it can be utilized by those who need it (e.g., those with mobility restrictions or living in remote locations).
- Advocacy should aim to support research specifically addressing sex- and gender-related factors on the course of IBD, on the lived experience of IBD, and how to best minimize disparities based on sex and/or gender—including non-binary and other genders. A strong knowledge translation focus on this research will inform clinicians and may positively affect the quality of care they provide.
- Clinical guidelines recommend screening people with IBD for mental health concerns as part of standard practice in IBD care, recognizing the prominent comorbidity and disease influence, but also noting the importance of having clinical pathways for care if positive. Psychological therapies used to treat anxiety and depression occurring in the context of IBD have been shown to significantly improve quality of life and reduce anxiety and depression for children and adults with IBD; there is less direct evidence in regard to the impact of antidepressant medication on mental health or disease outcomes in IBD.
- Crohn’s and Colitis Canada’s COVID-19 and IBD taskforce compiled knowledge and communicated with the IBD community through an expert-generated website and frequent webinars for a public audience. This work has set the stage for Crohn’s and Colitis Canada to inform the IBD community in the event of any future public health emergencies. Crohn’s and Colitis Canada should continue to advocate policy makers and health authorities during the pandemic for vulnerable, immunocompromised individuals with IBD.
- Additional resources to facilitate shared decision-making discussions are required. Shared decision-making needs to be promoted and facilitated for all aspects of healthcare for individuals with IBD. It is particularly important for communicating and making decisions with respect to cancer care. Shared decision-making should inform treatment choices and cancer risks for IBD, weighing the risks of untreated inflammatory disease against the small, but potentially serious, risks of extra-intestinal cancers with immunosuppressive therapies.
- Strategies to optimize the effectiveness of available therapies, such as introducing biologic therapy early in the course of Crohn’s disease, targeting normalization of objective markers of disease remission, and using therapeutic drug monitoring to guide treatment decisions with biologics have the potential to improve long-term prognosis and longevity of current medical treatment options.
- Advocacy for increased government investment in the development, implementation, and evaluation of evidence-based interventions and practices that improve access to IBD care, particularly for equity-deserving populations, is needed. Promising interventions and practices include, but are not limited to, increased specialist staffing in under resourced areas, augmented psychosocial supports, continued virtual health supports, and support for patient navigators.
- For future Impact of IBD in Canada reports, it is recommended that Crohn’s and Colitis Canada continue its important work of engaging patient and caregiver partners. Importantly, this community should be engaged at the beginning of the process such that they can help frame the research questions and inform which chapters should be included in the report.

## SUPPLEMENT SPONSORSHIP

This article appears as part of the supplement “The Impact of Inflammatory Bowel Disease in Canada in 2023”, sponsored by Crohn’s and Colitis Canada, and supported by Canadian Institutes of Health Research Project Scheme Operating Grant (Reference number PJT-162393).

gwad003_suppl_Supplementary_Material_1

gwad003_suppl_Supplementary_Material_2

## Data Availability

No new data were generated or analyzed in support of this review.
