# Correction to: The 2023 Impact of Inflammatory Bowel Disease in Canada: Executive Summary

**DOI:** 10.1093/jcag/gwad038

**Published:** 2023-11-13

Joseph W Windsor, M Ellen Kuenzig, Sanjay K Murthy, Alain Bitton, Charles N Bernstein, Jennifer L Jones, Kate Lee, Laura E Targownik, Juan-Nicolás Peña-Sánchez, Noelle Rohatinsky, Sara Ghandeharian, James H B Im, Tal Davis, Jake Weinstein, Quinn Goddard, Julia Gorospe, Eric I Benchimol, Gilaad G Kaplan, Impact of IBD in Canada Patient & Caregiver Partners; Impact of IBD in Canada Systematic Reviewers, The 2023 Impact of Inflammatory Bowel Disease in Canada: Executive Summary, Journal of the Canadian Association of Gastroenterology, Volume 6, Issue Supplement_2, September 2023, Pages S1–S8, https://doi.org/10.1093/jcag/gwad003

In the originally published version of this manuscript, author Julia Gorospe was inadvertently omitted from the author list.

Throughout the manuscript, the term ‘healthcare’ was given as ‘health care’.

In the following sentence, ‘writing group’ should have been given as ‘working group’:

“This paragraph is in the partners’ words and is not edited by the writing group of the article or the Steering Committee of the report.”

The manuscript has been corrected accordingly.

