# Supplementary material for: The 2023 Impact of Inflammatory Bowel Disease in Canada: Executive Summary
Source: J Can Assoc Gastroenterol. 2023 Jun 1;6(Suppl 2):S1–8. doi: 10.1093/jcag/gwad003 (PMC10478799; doi:10.1093/jcag/gwad003)
Supplement: gwad003_suppl_Supplementary_Material_1 [file gwad003_suppl_supplementary_material_1.docx]

**Supplement 1: Systematic Review Search Strategies**

Table 1.1: Access to Care inclusion and exclusion criteria

| **Inclusion Criteria** | **Exclusion Criteria** |
| --- | --- |
| - Observational study describing or quantifying any type of barrier to care for people with inflammatory bowel disease (IBD), Crohn’s disease (CD), or ulcerative colitis (UC); or - Observational study describing or quantifying the impact of barriers to care on any IBD-related outcomes (e.g., disease severity index, hospitalization, surgery, treatment-related outcome) in people with IBD, CD, or UC; or - Observational study describing availability of care for people with IBD, CD, or UC (e.g., availability of gastroenterologists, availability of IBD nurses or other allied health); or - Observational study describing regional variation in access to care, availability of care, or outcomes of care (e.g., disease severity index, hospitalization, surgery, treatment-related outcomes, etc) in people with IBD, CD, or UC; or - Qualitative study describing IBD (or CD or UC) patients’ or caregivers’ perspectives of access to care, availability of care, barriers to care; or - Observational study describing any aspect of telehealth or remote/virtual care (synchronous or asynchronous) in the care of IBD, CD, or UC patients; or - Qualitative study describing IBD/CD/UC patients’ or caregivers’ perspectives of telehealth or remote/virtual care (synchronous or asynchronous); or - Observational study describing any aspect of cultural barriers to care in people with IBD, CD, or UC, including the impact of cultural barriers on outcomes (e.g., disease severity, hospitalization, surgery, treatment-related outcomes, etc); or - Qualitative study describing cultural barriers to care or the importance/provision of culturally-appropriate care in people with IBD, CD, or UC; or - Observational study describing metrics for quality improvement, quality of care, quality indicators, etc in the care of people with IBD, CD, or UC; or - Qualitative study describing IBD, CD, or UC patients’ or caregivers’ perspectives of quality of care, quality indicators, quality improvement etc; or - Qualitative study describing IBD, CD, or UC patients’ or caregivers’ perspectives of integrated/multidisciplinary care models; or - Observational study describing availability of integrated/multidisciplinary care for people with IBD, CD, or UC or the outcomes of integrated/multidisciplinary care; or - Qualitative or observational study describing patient perspectives of shared decision making or IBD outcomes associated with shared decision making or incorporating patient preferences; or - Clinical practice guideline outlining the use of telehealth or remote/virtual care in IBD, CD, or UC; or - Clinical practice guideline describing best practices for providing culturally-competent care for IBD, CD, or UC; or - Clinical practice guideline describing integrated models of care for IBD, CD, or UC patients; or - Clinical practice guidelines describing approaches for integrating shared decision making or patient preferences in patients with IBD, CD, or UC (or their caregivers); or - Review article synthesizing evidence on any of the above topics. | - Conference abstract - Study is not in English - Does not include people with IBD, CD, or UC (or their caregivers) - Letter, editorial, or commentary not including any original data - Study does not describe barriers to care, quality of care, availability of care, or integrated models of care - Case report or series including <5 IBD patients |

Table 1.2a: Access to Care search terms (MEDLINE), performed June 8, 2022

| **Ovid MEDLINE: Epub Ahead of Print, In-Process & Other Non-Indexed Citations, Ovid MEDLINE® Daily and Ovid MEDLINE® <1946-Present>** | | |
| --- | --- | --- |
| 1 | exp Inflammatory Bowel Diseases/ | 91743 |
| 2 | exp Colitis, Ulcerative/ | 38540 |
| 3 | exp Crohn Disease/ | 42650 |
| 4 | exp Proctitis/ | 3175 |
| 5 | exp Enteritis/ | 14200 |
| 6 | ("Inflammatory bowel disease$" or ulcerative colitis or Crohn$ or IBD or ileocolitis or ileitis or pancolitis or proctitis).mp. | 129753 |
| 7 | or/1-6 [Set 1: IBD] | 144186 |
| 8 | exp health services accessibility/ or healthcare disparities/ or "health care quality, access, and evaluation"/ or health services research/ | 171804 |
| 9 | "Quality of Health Care"/ | 76691 |
| 10 | Delayed Diagnosis/ | 7883 |
| 11 | (access$ adj5 health$).ti,ab,kf. | 54505 |
| 12 | (quality adj5 care).ti,ab,kf. | 112265 |
| 13 | (delay$ adj5 diagnos$).ti,ab,kf. | 39514 |
| 14 | (equ$ adj5 access$ adj5 care).ti,ab,kf. | 2210 |
| 15 | culturally competent care/ | 2034 |
| 16 | (cultur$ adj5 competen$).ti,ab,kf. | 7883 |
| 17 | "Delivery of Health Care, Integrated"/ | 13961 |
| 18 | (integrated adj3 care).ti,ab,kf. | 13397 |
| 19 | exp Nurse Practitioners/ | 18796 |
| 20 | practice patterns, nurses'/ or practice patterns, physicians'/ | 68656 |
| 21 | ((IBD or "inflammatory bowel disease" or gastroenterol$) adj3 nurs$).ti,ab,kf. | 483 |
| 22 | telemedicine/ or remote consultation/ | 38175 |
| 23 | ((virtual or video$) adj3 (gastroenterolog$ or outpatient or follow-up or consult$)).ti,ab,kf. | 2680 |
| 24 | (telehealth or telemedicine).ti,ab,kf. | 27969 |
| 25 | Nutritionists/ | 1614 |
| 26 | dieti#ian$.ti,ab,kf. | 9312 |
| 27 | (psychologist$ or psychiatrist$ or social worker$).ti,ab,kf. | 52080 |
| 28 | Social Workers/ | 946 |
| 29 | (integrat$ adj3 (care or healthcare)).ti,ab,kf. | 22914 |
| 30 | (model$ adj3 (care or healthcare)).ti,ab,kf. | 30503 |
| 31 | health resources/ or health workforce/ | 28462 |
| 32 | ((number or supply or availab$) adj5 gastroenterolog$).ti,ab,kf. | 258 |
| 33 | exp Patient-Centered Care/ | 23233 |
| 34 | (patient-centred or patient-centered or patient-focused).ti,ab,kf. | 31179 |
| 35 | (multidisciplinary adj (care or healthcare)).ti,ab,kf. | 4556 |
| 36 | nurse-patient relations/ or physician-patient relations/ | 110374 |
| 37 | (patient$ adj3 (physician$ or gastroenterolg$ or nurse$) adj3 (relation$ or partner$)).ti,ab,kf. | 8831 |
| 38 | Quality Assurance, Health Care/ | 56803 |
| 39 | exp Quality Improvement/ | 32071 |
| 40 | exp Quality Indicators, Health Care/ | 24281 |
| 41 | (quality adj5 (care or healthcare)).ti,ab,kf. | 123625 |
| 42 | (shared adj3 (care or healthcare or decision$)).ti,ab,kf. | 15690 |
| 43 | Patient Access to Records/ | 1152 |
| 44 | (access$ adj5 (information or record$ or chart$)).ti,ab,kf. | 25042 |
| 45 | Decision Making, Shared/ | 1598 |
| 46 | or/8-45 | 880280 |
| 47 | 7 and 46 | 3089 |
| 48 | limit 47 to yr="2018-current" | 1322 |
| 49 | limit 48 to english language | 1300 |

Table 1.2b: Access to Care search terms (EMBASE), performed June 8, 2022

| **Embase Classic+Embase <1947 to 2022 June 07>** | | |
| --- | --- | --- |
| 1 | Inflammatory bowel disease/ | 45728 |
| 2 | exp Crohn disease/ | 105793 |
| 3 | exp ulcerative colitis/ | 89879 |
| 4 | proctitis/ | 7604 |
| 5 | ileitis/ | 7372 |
| 6 | pancolitis/ | 2234 |
| 7 | proctocolitis/ | 1355 |
| 8 | ("Inflammatory bowel disease*" or ulcerative colitis or Crohn* or IBD or ileocolitis or ileitis or pancolitis or proctitis).af. | 238439 |
| 9 | or/1-8 [Set 1: IBD] | 239248 |
| 10 | exp health care access/ | 85373 |
| 11 | health care disparity/ | 19857 |
| 12 | health care quality/ or health equity/ | 266314 |
| 13 | health services research/ | 35277 |
| 14 | delayed diagnosis/ | 15912 |
| 15 | (access$ adj5 health$).ti,ab,kf. | 68677 |
| 16 | (quality adj5 care).ti,ab,kf. | 154325 |
| 17 | (delay$ adj5 diagnos$).ti,ab,kf. | 62737 |
| 18 | (equ$ adj5 access$ adj5 care).ti,ab,kf. | 2849 |
| 19 | exp transcultural care/ | 6750 |
| 20 | (cultur$ adj5 competen$).ti,ab,kf. | 9437 |
| 21 | integrated health care system/ | 12637 |
| 22 | (integrated adj3 care).ti,ab,kf. | 18176 |
| 23 | nurse practitioner/ or acute care nurse practitioner/ or pediatric nurse practitioner/ | 27335 |
| 24 | clinical practice/ | 329680 |
| 25 | ((IBD or "inflammatory bowel disease" or gastroenterol$) adj3 nurs$).ti,ab,kf. | 1140 |
| 26 | exp telemedicine/ | 60647 |
| 27 | ((virtual or video$) adj3 (gastroenterolog$ or outpatient or follow-up or consult$)).ti,ab,kf. | 3772 |
| 28 | (telehealth or telemedicine).ti,ab,kf. | 36622 |
| 29 | dietitian/ | 15092 |
| 30 | dieti#ian$.ti,ab,kf. | 17154 |
| 31 | (psychologist$ or psychiatrist$ or social worker$).ti,ab,kf. | 86892 |
| 32 | social worker/ | 15295 |
| 33 | psychologist/ | 17195 |
| 34 | physician/ or gastroenterologist/ or psychiatrist/ | 383600 |
| 35 | (integrat$ adj3 (care or healthcare)).ti,ab,kf. | 31772 |
| 36 | (model$ adj3 (care or healthcare)).ti,ab,kf. | 42772 |
| 37 | health care planning/ | 109346 |
| 38 | exp health workforce/ | 2530 |
| 39 | ((number or supply or availab$) adj5 gastroenterolog$).ti,ab,kf. | 546 |
| 40 | patient care/ or collaborative care team/ or patient decision making/ or shared medical appointment/ | 344194 |
| 41 | (patient-centred or patient-centered or patient-focused).ti,ab,kf. | 42997 |
| 42 | (multidisciplinary adj (care or healthcare)).ti,ab,kf. | 7479 |
| 43 | professional-patient relationship/ or doctor patient relationship/ or nurse patient relationship/ | 53261 |
| 44 | (patient$ adj3 (physician$ or gastroenterolg$ or nurse$) adj3 (relation$ or partner$)).ti,ab,kf. | 10236 |
| 45 | total quality management/ | 79468 |
| 46 | (quality adj5 (care or healthcare)).ti,ab,kf. | 169355 |
| 47 | (shared adj3 (care or healthcare or decision$)).ti,ab,kf. | 22313 |
| 48 | shared decision making/ | 11201 |
| 49 | patient right/ or patient autonomy/ | 20439 |
| 50 | (access$ adj5 (information or record$ or chart$)).ti,ab,kf. | 33668 |
| 51 | or/10-50 | 1916318 |
| 52 | 9 and 51 | 17278 |
| 53 | limit 52 to yr="2018-current" | 7120 |
| 54 | limit 53 to english language | 7003 |
| 55 | limit 54 to conference abstract | 3511 |
| 56 | 54 not 55 | 3492 |

Table 1.2c: Access to Care search terms (CINAHL), performed June 8, 2022

| **CINAHL** | | | |
| --- | --- | --- | --- |
| S1 | (MH "Inflammatory Bowel Diseases+") | Search modes - Boolean/Phrase | 18051 |
| S2 | (MH “Crohn Disease”) | Search modes - Boolean/Phrase | 7763 |
| S3 | (MH “Colitis, Ulcerative”) | Search modes - Boolean/Phrase | 5991 |
| S4 | (MH “Ileitis+”) | Search modes - Boolean/Phrase | 346 |
| S5 | TX ("Inflammatory bowel disease*" or ulcerative  colitis or Crohn* or IBD or ileocolitis or ileitis or pancolitis or proctitis) | Search modes - Boolean/Phrase | 32334 |
| S6 | S1 or S2 or S3 or S4 or S5 | Search modes - Boolean/Phrase | 33143 |
| S7 | (MH "Healthcare Disparities") OR (MH  "Health Care Delivery, Integrated") OR (MH "Health Resource Allocation") OR (MH "Health Resource Utilization") OR (MH "Health Services Accessibility+") | Search modes – Boolean/Phrase | 147644 |
| S8 | (MH “Quality of Health Care”) | Search modes – Boolean/Phrase | 81,228 |
| S9 | (MH “Diagnosis, Delayed”) | Search modes – Boolean/Phrase | 4992 |
| S10 | TX (“access to care” or “access to healthcare” or “access to health” or “accessibility of health” or “accessibility of care”) | Search modes – Boolean/Phrase | 45,997 |
| S11 | TX (“quality of care” or “quality of healthcare” or “quality of health care”) | Search modes – Boolean/Phrase | 166600 |
| S12 | TX (“healthcare quality” or “health care quality”) | Search modes – Boolean/Phrase | 15517 |
| S13 | TX ("accessible care" or "accessible healthcare" or  "accessible health care" or "accessing care" or "accessing healthcare" or "accessing health care") | Search modes – Boolean/Phrase | 6027 |
| S14 | TX (“healthcare accessibility” or “healthcare accessibility” or “care accessibility”) | Search modes – Boolean/Phrase | 565 |
| S15 | TX ("diagnostic delay" or "delayed diagnosis" or  "diagnostic lag") | Search modes – Boolean/Phrase | 5680 |
| S16 | TX ("equal access" or "equitable access" or  "health equity") | Search modes – Boolean/Phrase | 18905 |
| S17 | (MH "Cultural Competence") OR (MH  "Transcultural Care") | Search modes – Boolean/Phrase | 13763 |
| S18 | TX ("culturally competent care" or "culturally  appropriate care" or "culturally competent healthcare" or "culturally competent health care" or "culturally appropriate health care" or "culturally appropriate healthcare") | Search modes – Boolean/Phrase | 3150 |
| S19 | (MH "Health Care Delivery, Integrated") | Search modes – Boolean/Phrase | 13618 |
| S20 | TX ("integrated care" or "integrated healthcare" or  "integrated health care") | Search modes – Boolean/Phrase | 17818 |
| S21 | (MH "Pediatric Nurse Practitioners") OR (MH  "Nurse Practitioners") | Search modes – Boolean/Phrase | 22106 |
| S22 | (MH “Practice Patterns”) | Search modes – Boolean/Phrase | 13156 |
| S23 | TX ("inflammatory bowel disease nurs*" or "IBD  nurs*" or "gastroenterolog* nurs") | Search modes – Boolean/Phrase | 381 |
| S24 | (MH "Telemedicine") OR (MH "Remote  Consultation") OR (MH "Telenursing") | Search modes – Boolean/Phrase | 19362 |
| S25 | TX (“virtual gastroneterolg*” or “virtual specialist visit*” or “virtual specialist consult*” or “virtual appointment*” or “virtual outpatient” or “virtual clinic”) | Search modes – Boolean/Phrase | 883 |
| S26 | TX ("remote gastroenterolog*" or  "remote specialist visit*" or "remote specialist consult*" or "remote appointment*" or "remote outpatient" or "remote clinic*") | Search modes – Boolean/Phrase | 482 |
| S27 | (MH “Dietitians”) | Search modes – Boolean/Phrase | 5739 |
| S28 | (MH “Social Workers”) | Search modes – Boolean/Phrase | 10344 |
| S29 | (MH “Psychologists”) | Search modes – Boolean/Phrase | 4753 |
| S30 | (MH “Psychiatrists”) | Search modes – Boolean/Phrase | 3513 |
| S31 | TX (dietician* or dietitian* or "social work*" or  psychologist* or psychiatr*) | Search modes – Boolean/Phrase | 669928 |
| S32 | TX ("healthcare model*" or "health care model*" or  "model* of care" or "model* of health care" or "model* of health") | Search modes – Boolean/Phrase | 34829 |
| S33 | (MH "Health Resource Allocation") OR (MH  "Health Services Needs and Demand") | Search modes – Boolean/Phrase | 36127 |
| S34 | TX ("number of gastroenterolog*" or  "availability of gastroenterolog*" or "availability of nurs*" or "number of nurs*") | Search modes – Boolean/Phrase | 10682 |
| S35 | (MH "Patient Centered Care") | Search modes – Boolean/Phrase | 33907 |
| S36 | TX ("patient centred care" or "patient centered care" or "patient focused care") | Search modes – Boolean/Phrase | 45075 |
| S37 | TX ("multidisciplinary care" or "multidisciplinary  health") | Search modes – Boolean/Phrase | 53,353 |
| S38 | (MH “Nurse-Patient Relations”) OR (MH “Physician-Patient Relations”) | Search modes – Boolean/Phrase | 64527 |
| S39 | TX ("patient-physician relation*" or "patient-nurse  relation*" or "patient- gastroenterolog* relation*" or "patient partner*" or "partner* patient* or "physician-patient relation* or "nurse-patient relation*" or "gastroenterolog* patient relation*" or "physician- patient relation*") | Search modes – Boolean/Phrase | 71048 |
| S40 | (MH "Quality Assurance") OR (MH "Quality of Care  Research") OR (MH "Quality of Health Care") OR (MH "Quality Improvement+") OR (MH "Quality Assessment") | Search modes – Boolean/Phrase | 171786 |
| S41 | (MH “Decision Making, Shared”) | Search modes – Boolean/Phrase | 2680 |
| S42 | TX ("shared decision*" or "shared care" or "shared  healthcare" or "shared health") | Search modes – Boolean/Phrase | 17357 |
| S43 | (MH "Patient Access to Records") | Search modes – Boolean/Phrase | 1086 |
| S44 | TX ("access to information" or "access to  health information" or "access to record*" or "access to health record*" or "access to chart" or "accessibility of health information" or "accessibility of information" or "access to chart*” or “access to medical chart*”) | Search modes – Boolean/Phrase | 36774 |
| S45 | S7 OR S8 OR S9 OR S10 OR S11 OR S12 OR S13 OR S14 OR S15 OR S16 OR S17 OR S18 OR S19 OR S20 OR S21 OR S22 OR S23 OR S24 OR S25 OR S26 OR S27 OR S28 OR S29 OR S30 OR S31 OR S32 OR S33 OR S34 OR S35 OR S36 OR S37 OR S38 OR S39 OR S40 OR S41 OR S42 OR S43 OR S44 | Search modes – Boolean/Phrase | 1258775 |
| S46 | S6 AND S45 | Search modes – Boolean/Phrase | 4732 |
| S47 | S6 AND S45 | Limiters - Published Date: 20180101-; Language: English Search modes - Boolean/Phrase | 1472 |


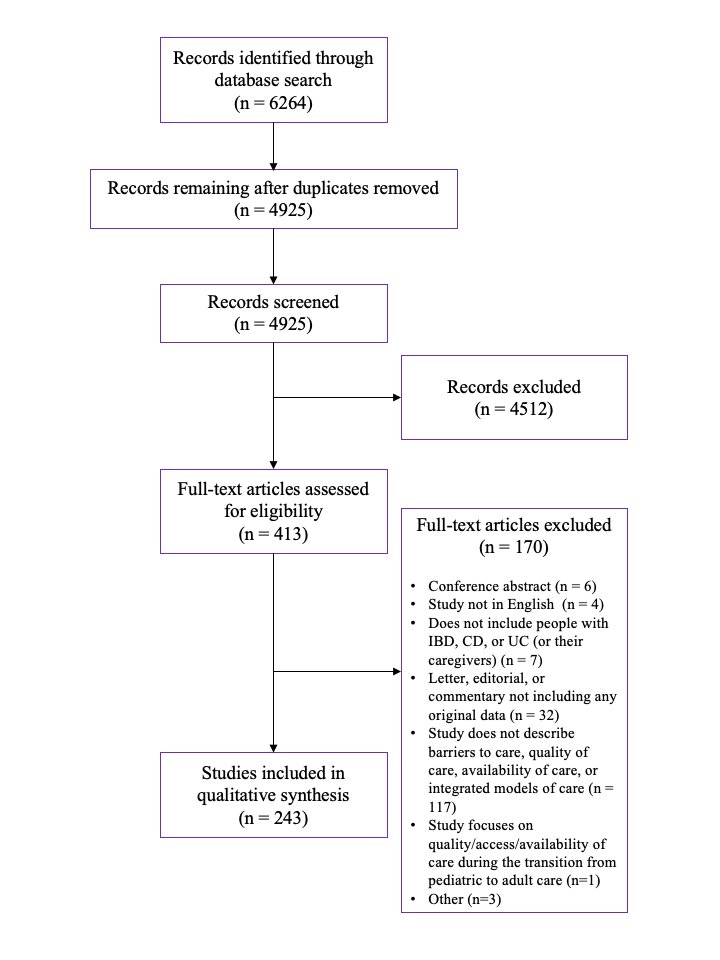


Figure 1: Flowchart detailing study selection for the Access to Care systematic review

Table 2.1: Cancer inclusion and exclusion criteria

| **Inclusion Criteria** | **Exclusion Criteria** |
| --- | --- |
| - Part 1: Epidemiology   - Population-based observational study describing the incidence, prevalence, or outcomes of any type of cancer in people with inflammatory bowel disease (IBD), Crohn’s disease (CD), or ulcerative colitis (UC); or   - Population-based observational study comparing the incidence, prevalence, or outcomes of any type of cancer in people with and without IBD, CD, or UC. Also include studies comparing the risk of any type of cancer across IBD subtypes; or   - Systematic or narrative review describing the incidence of any type of cancer in people with IBD, CD, or UC or comparing the risk of cancer in people with and without IBD.   *Note: Population-based studies are those that provide estimates of incidence or prevalence of cancer among all individuals living within a geographically well-defined political jurisdiction.*   - Part 2: Impact of treatment   - Observational study comparing the risk of any type of cancer in people with IBD, Crohn’s disease, or ulcerative colitis across therapies used to treat IBD, including biologics (anti-TNF, anti-integrin, anti-interleukin), immunomodulators (methotrexate, azathioprine/6-MP, cyclosporine), mesalamine and steroids; or   - Analysis of randomized controlled data of an IBD treatment looking at the risk of any type of cancer as an adverse event during the clinical trial program or during post-marketing surveillance; or   - Systematic or narrative review summarizing the association between IBD therapy and the risk of any type of cancer in people with IBD.   *Note: The comparison group must include people with IBD not on therapy. Studies that include a non-IBD control group should be excluded.*   - Part 3: Prevention   - Population-based study describing rates of cancer screening in people with IBD, CD, or UC; or   - Population-based study comparing rates of cancer screening in people with and without IBD, CD, or UC; or   - Clinical practice guidelines describing recommendations for cancer screening in people with IBD; or   - Systematic or narrative review describing rates of cancer screening in people with IBD, CD, or UC; or   - Systematic or narrative review comparing rates of cancer screening in people with and without IBD, CD, or UC; or   - Economic evaluation (e.g., cost-benefit or cost-effectiveness analysis) of cancer screening recommendations in people with IBD, CD, or UC; or   - Observational study or randomized controlled trial comparing the efficacy or effectiveness of approaches to cancer screening in people with IBD, CD, or UC or comparing the efficacy/effectiveness of approaches in people with and without IBD.   *Note: Include the following types of cancer screening: endoscopy (including studies describing random biopsies or chromoendoscopy), fecal occult blood tests, pap smears, skin cancer screening, mammography, cholangiopancreatography (ERCP or MRCP), other diagnostic imaging, or any additional forms of cancer screening tests.* | - Study is not in English - Conference abstract - Letter, editorial, or commentary not presenting original data - Case series or report including <5 IBD patients - Does not include people with IBD - Basic or translational science study - Describes the epidemiology of cancer in IBD patients but is not a population-based study - Compares the risk of cancer in IBD patients to people without IBD but is not a population-based study - Assesses the impact of IBD therapy on cancer risk but does not include a comparator group - Assesses the impact of IBD therapy on cancer risk but compares IBD patients on therapy to people without IBD - Describes rates of cancer screening in IBD patients but is not a population-based study or systematic/narrative review - Compares rates of cancer screening in people with and without but is not a population-based study or systematic/narrative review - Clinical practice guideline describing recommendations for cancer screening in people without IBD - Economic evaluation of cancer screening but not specific to people with IBD, CD, or UC - Study evaluating efficacy/effectiveness of an approach to cancer screening but not in people with IBD or comparing the efficacy/effectiveness of an approach in people with and without IBD |

Table 2.2a: Cancer search terms (MEDLINE), performed May 9, 2022

| **Ovid MEDLINE: Epub Ahead of Print, In-Process & Other Non-Indexed Citations, Ovid MEDLINE® Daily and Ovid MEDLINE® <1946-Present>** | | |
| --- | --- | --- |
| 1 | exp Inflammatory Bowel Diseases/ | 91323 |
| 2 | exp Colitis, Ulcerative/ | 38388 |
| 3 | exp Crohn Disease/ | 42487 |
| 4 | exp Proctitis/ | 3168 |
| 5 | exp Enteritis/ | 14163 |
| 6 | ("Inflammatory bowel disease*" or ulcerative colitis or Crohn* or IBD or ileocolitis or ileitis or pancolitis or proctitis).mp. | 129108 |
| 7 | or/1-6 [Set 1: IBD] | 143509 |
| 8 | exp Neoplasms/ | 3684610 |
| 9 | (cancer* or carcinoma or neoplas* or lymphoma or leukemia or melanoma or cholangiocarcinoma or hepatoma or tumo#r).ti,ab,kf. | 3194243 |
| 10 | or/8-9 [Set 2: Cancer] | 4497138 |
| 11 | Epidemiology/ | 12552 |
| 12 | incidence/ | 292623 |
| 13 | Prevalence/ | 330290 |
| 14 | Death/ | 19113 |
| 15 | exp Mortality/ | 418095 |
| 16 | Survival/ | 4907 |
| 17 | (epidemiolog* or inciden* or prevalen*).ti,ab,kf. | 2117604 |
| 18 | exp Prognosis/ | 1842807 |
| 19 | (temporal trend* or time trend* or secular trend*).ti,ab,kf. | 25388 |
| 20 | (death or mortality or survival).ti,ab,kf. | 2385325 |
| 21 | prognos*.ti,ab,kf. | 727526 |
| 22 | or/11-21 [Set 3: Epidemiology] | 5722979 |
| 23 | Tumor Necrosis Factor-alpha/ or Tumor Necrosis Factor Inhibitors/ | 134269 |
| 24 | Infliximab/ | 11521 |
| 25 | Adalimumab/ | 6457 |
| 26 | Certolizumab Pegol/ | 705 |
| 27 | (infliximab or adalimumab or certolizumab or golimumab).ti,ab,kf. | 19238 |
| 28 | vedolizumab.ti,ab,kf. | 1335 |
| 29 | Ustekinumab/ | 1506 |
| 30 | ustekinumab.ti,ab,kf. | 2467 |
| 31 | Mesalamine/ or Sulfasalazine/ | 7496 |
| 32 | (aminosalicylic acid or aminosalicylate).ti,ab,kf. | 6509 |
| 33 | (mesalazine or mesalamine).ti,ab,kf. | 2784 |
| 34 | (olsalazine or balsalazide or sulfasalazine).ti,ab,kf. | 3877 |
| 35 | exp Adrenal Cortex Hormones/ | 415549 |
| 36 | (prednisone or prednisolone or corticosteroid* or steroid*).ti,ab,kf. | 397374 |
| 37 | Azathioprine/ or Mercaptopurine/ | 20233 |
| 38 | (mercaptopurine or 6*MP or 6MP or azathioprine or thiopurine).ti,ab,kf. | 21192 |
| 39 | Methotrexate/ | 40180 |
| 40 | methotrexate.ti,ab,kf. | 45267 |
| 41 | immunomodulator$.ti,ab,kf. | 55472 |
| 42 | Janus Kinase Inhibitors/ | 1052 |
| 43 | tofacitinib.ti,ab,kf. | 2090 |
| 44 | Cyclosporine/ | 30199 |
| 45 | cyclosporine.ti,ab,kf. | 31873 |
| 46 | or/23-45 [Set 4: Therapeutics] | 958945 |
| 47 | "Early Detection of Cancer"/ | 33658 |
| 48 | exp Endoscopy, Gastrointestinal/ | 97333 |
| 49 | Cholangiopancreatography, Magnetic Resonance/ or Cholangiopancreatography, Endoscopic Retrograde/ | 19699 |
| 50 | Magnetic Resonance Imaging/ | 453208 |
| 51 | Tomography, X-Ray Computed/ | 408614 |
| 52 | Papanicolaou Test/ | 7071 |
| 53 | Mass Screening/ | 113366 |
| 54 | Mammography/ | 32160 |
| 55 | (cancer adj3 (screen$ or detect$ or missed or early or post-colonoscop$ or surveillance)).ti,ab,kf. | 135447 |
| 56 | Occult Blood/ | 6088 |
| 57 | (colonoscop$ or (lower adj endoscop$) or sigmoidoscop$).ti,ab,kf. | 37702 |
| 58 | f?ecal occult blood.ti,ab,kf. | 4369 |
| 59 | Cholangiopancreatography.ti,ab,kf. | 12196 |
| 60 | (MR or magnetic resonance or CT or computerized tomography).ti,ab,kf. | 873273 |
| 61 | (pap$ adj (test or smear)).ti,ab,kf. | 9302 |
| 62 | mammogra$.ti,ab,kf. | 35127 |
| 63 | or/47-62 [Set 5: Screening] | 1626449 |
| 64 | 7 and 10 and (22 or 46 or 63) | 13298 |
| 65 | limit 64 to yr="2018-current" | 3158 |
| 66 | limit 65 to english language | 3089 |
| 67 | 66 not ((exp animal/ or exp invertebrate/ or animal experiment/ or animal model/ or exp plant/ or exp fungus/) not exp human/) | 2962 |

Table 2.2b: Cancer search terms (EMBASE), performed May 9, 2022

| **Embase Classic+Embase <1947 to 2022 May 06>** | | |
| --- | --- | --- |
| 1 | Inflammatory bowel disease/ | 45077 |
| 2 | exp Crohn disease/ | 105411 |
| 3 | exp ulcerative colitis/ | 89491 |
| 4 | proctitis/ | 7581 |
| 5 | ileitis/ | 7344 |
| 6 | pancolitis/ | 2209 |
| 7 | proctocolitis/ | 1353 |
| 8 | ("Inflammatory bowel disease*" or ulcerative colitis or Crohn* or IBD or ileocolitis or ileitis or pancolitis or proctitis).af. | 237168 |
| 9 | or/1-8 [Set 1: IBD] | 237978 |
| 10 | exp malignant neoplasm/ | 4132913 |
| 11 | (cancer* or carcinoma or neoplas* or lymphoma or leukemia or melanoma or cholangiocarcinoma or hepatoma or tumo#r).ti,ab,kf. | 4506184 |
| 12 | or/10-11 [Set 2: Cancer] | 5485189 |
| 13 | epidemiology/ or cancer epidemiology/ | 288996 |
| 14 | cancer incidence/ or incidence/ | 583547 |
| 15 | exp prevalence/ | 891497 |
| 16 | death/ | 327190 |
| 17 | mortality/ or cancer mortality/ or exp mortality rate/ or premature mortality/ | 1072870 |
| 18 | survival rate/ or exp cancer survival/ or survival/ | 994282 |
| 19 | exp prognosis/ | 879004 |
| 20 | (epidemiolog* or inciden* or prevalen*).ti,ab,kf. | 3037837 |
| 21 | trend study/ | 45934 |
| 22 | (temporal trend* or time trend* or secular trend*).ti,ab,kf. | 34344 |
| 23 | (death or mortality or survival).ti,ab,kf. | 3527971 |
| 24 | prognos*.ti,ab,kf. | 1133697 |
| 25 | or/13-24 [Set 3: Epidemiology] | 7354687 |
| 26 | tumor necrosis factor/ | 188713 |
| 27 | tumor necrosis factor antibody/ | 5745 |
| 28 | infliximab/ | 57440 |
| 29 | adalimumab/ | 40129 |
| 30 | certolizumab pegol/ | 8114 |
| 31 | golimumab/ | 8702 |
| 32 | (infliximab or adalimumab or certolizumab or golimumab).ti,ab,kf. | 43372 |
| 33 | vedolizumab/ | 5844 |
| 34 | vedolizumab.ti,ab,kf. | 3980 |
| 35 | ustekinumab/ | 9961 |
| 36 | ustekinumab.ti,ab,kf. | 5672 |
| 37 | mesalazine/ | 20404 |
| 38 | salazosulfapyridine/ | 28032 |
| 39 | (aminosalicylic acid or aminosalicylate).ti,ab,kf. | 6108 |
| 40 | (mesalazine or mesalamine).ti,ab,kf. | 5590 |
| 41 | (olsalazine or balsalazide or sulfasalazine).ti,ab,kf. | 6528 |
| 42 | olsalazine/ | 1501 |
| 43 | balsalazide/ | 961 |
| 44 | glucocorticoid/ or corticosteroid/ or prednisone/ | 542750 |
| 45 | (prednisone or prednisolone or corticosteroid* or steroid*).ti,ab,kf. | 619725 |
| 46 | azathioprine/ or mercaptopurine/ | 124998 |
| 47 | (mercaptopurine or 6*MP or 6MP or azathioprine or thiopurine).ti,ab,kf. | 38230 |
| 48 | methotrexate/ | 200298 |
| 49 | methotrexate.ti,ab,kf. | 78054 |
| 50 | immunomodulator$.ti,ab,kf. | 83942 |
| 51 | janus kinase inhibitor/ or tofacitinib/ | 10611 |
| 52 | tofacitinib.ti,ab,kf. | 4436 |
| 53 | cyclosporine/ | 22472 |
| 54 | cyclosporine.ti,ab,kf. | 48128 |
| 55 | or/26-54 [Set 4: Therapeutics] | 1454920 |
| 56 | early cancer diagnosis/ | 10740 |
| 57 | exp intestine endoscopy/ | 120188 |
| 58 | cholangiography/ or endoscopic retrograde cholangiopancreatography/ or magnetic resonance cholangiopancreatography/ | 62872 |
| 59 | nuclear magnetic resonance imaging/ or magnetic resonance enterography/ | 903782 |
| 60 | computer assisted tomography/ or computed tomography enterography/ | 822110 |
| 61 | papanicolaou test/ | 20035 |
| 62 | mass screening/ or cancer screening/ | 151033 |
| 63 | exp mammography/ | 63963 |
| 64 | occult blood test/ | 6337 |
| 65 | (cancer adj3 (screen$ or detect$ or missed or early or post-colonoscop$ or surveillance)).ti,ab,kf. | 202221 |
| 66 | (colonoscop$ or (lower adj endoscop$) or sigmoidoscop$).ti,ab,kf. | 75063 |
| 67 | f?ecal occult blood.ti,ab,kf. | 6707 |
| 68 | Cholangiopancreatography.ti,ab,kf. | 17839 |
| 69 | (MR or magnetic resonance or CT or computerized tomography).ti,ab,kf. | 1275267 |
| 70 | (pap$ adj (test or smear)).ti,ab,kf. | 13511 |
| 71 | mammogra$.ti,ab,kf. | 47515 |
| 72 | or/56-71 [Set 5: Screening] | 2583762 |
| 73 | 9 and 12 and (25 or 55 or 72) | 32302 |
| 74 | limit 73 to yr="2018-current" | 10192 |
| 75 | limit 74 to english language | 10051 |
| 76 | limit 75 to conference abstract | 3651 |
| 77 | 75 not 76 | 6400 |
| 78 | 77 not ((exp animal/ or exp invertebrate/ or nonhuman/ or animal experiment/ or animal tissue/ or animal model/ or exp plant/ or exp fungus/) not (exp human/ or human tissue/)) | 6034 |


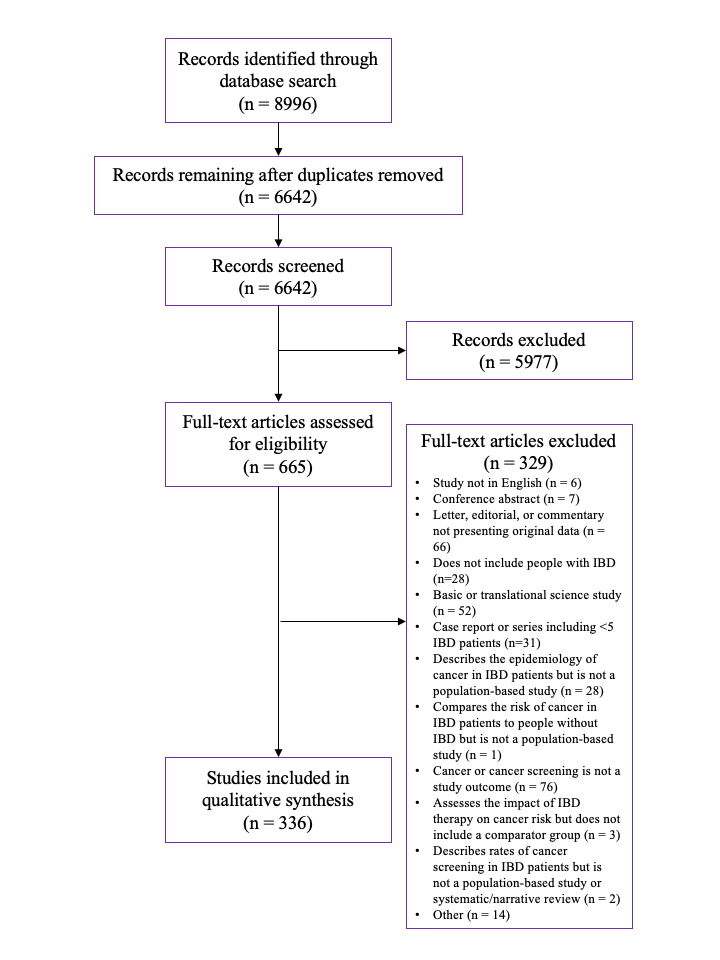


Figure 2: Flowchart detailing study selection for the Cancer systematic review

Table 3.1: Costs (Direct and Indirect Costs) inclusion and exclusion criteria

| **Inclusion Criteria** | **Exclusion Criteria** |
| --- | --- |
| - Observational study describing direct or indirect healthcare costs for people living with inflammatory bowel disease (IBD), Crohn’s disease (CD), or ulcerative colitis (UC); or - Observational study comparing direct or indirect healthcare costs for people with and without IBD, CD, or UC; or - Systematic review article describing direct or indirect health care costs for people with IBD; or - Qualitative study describing patients’ or caregivers’ experiences related to direct or indirect costs related to IBD; or - Qualitative study describing financial barriers to accessing care or therapy, including but not limited to pharmaceuticals, complementary and alternative therapies, supplements, dietician, psychologist, or other allied healthcare providers; or - Observational study identifying factors associated with high costs (direct or indirect).   *Direct healthcare costs: Healthcare expenditures for medically necessary services and treatments, paid for by public and private payers, including hospital-based care, outpatient physician consultations, prescription medications, diagnostic tests, diagnostic and therapeutic procedures, complex continuing care, and home care. Direct cost calculations can take the perspective of the public payer (i.e., government-covered drugs and services) or all third-party payers (i.e., government, private insurers, and other health plans).*  *Indirect healthcare costs: Costs borne by individuals and society that are not covered by third party payers, such as lost productivity due to illness and disability, premature retirement, premature death, lost productivity of caregivers, and out-of-pocket costs.*  *Note: Include studies where individuals with IBD are included in studies involving people with other chronic health conditions.* | - Study is not in English - Conference abstract - Letter, editorial, or commentary not presenting original data - Does not include people with IBD - Does not assess costs (direct or indirect) - Case report or series including <5 IBD patients - Narrative review (review article without systematic review) - Economic evaluation assessing cost-effectiveness, cost-benefit, or similar of an intervention |

Table 3.2a: Costs (Direct and Indirect Costs) search terms (MEDLINE), performed May 9, 2022

| **Ovid MEDLINE: Epub Ahead of Print, In-Process & Other Non-Indexed Citations, Ovid MEDLINE® Daily and Ovid MEDLINE® <1946-Present>** | | |
| --- | --- | --- |
| 1 | exp Inflammatory Bowel Diseases/ | 91323 |
| 2 | exp Colitis, Ulcerative/ | 38388 |
| 3 | exp Crohn Disease/ | 42487 |
| 4 | exp Proctitis/ | 3168 |
| 5 | exp Enteritis/ | 14163 |
| 6 | ("Inflammatory bowel disease$" or ulcerative colitis or Crohn$ or IBD or ileocolitis or ileitis or pancolitis or proctitis).af. | 131238 |
| 7 | or/1-6 [Set 1: IBD] | 145490 |
| 8 | "costs and cost analysis"/ or "cost of illness"/ or exp health care costs/ or health expenditures/ | 158974 |
| 9 | ((direct or health$ or indirect or medication$ or therap$ or surg$ or caregiv$ or prescription$ or hospital$ or drug$) adj5 (cost$ or expen$ or spending or payment$)).ti,ab,kf. | 181131 |
| 10 | "out-of-pocket".ti,ab,kf. | 7243 |
| 11 | absenteeism/ or presenteeism/ | 9894 |
| 12 | (absenteeism or presenteeism).ti,ab,kf. | 7518 |
| 13 | ((absent$ or miss$) adj5 (work or employment)).ti,ab,kf. | 4592 |
| 14 | (financial adj (impact or toxicity or distress or burden or hardship or effect or difficult$)).ti,ab,kf. | 11726 |
| 15 | (economic adj (burden or impact or implication$ or hardship$ or difficult$)).ti,ab,kf. | 28474 |
| 16 | friction cost.ti,ab,kf. | 111 |
| 17 | material hardship$.ti,ab,kf. | 229 |
| 18 | (societ$ adj cost$).ti,ab,kf. | 2582 |
| 19 | labo?r market.ti,ab,kf. | 5263 |
| 20 | ((loss or lost) adj3 productivity).ti,ab,kf. | 4458 |
| 21 | deprivation.ti,ab,kf. | 75262 |
| 22 | poverty.ti,ab,kf. | 30801 |
| 23 | bankrupt$.ti,ab,kf. | 772 |
| 24 | sick leave/ | 6504 |
| 25 | or/8-24 [Set 2: Costs of Care] | 438687 |
| 26 | 7 and 25 | 2039 |
| 27 | limit 26 to yr="2018-Current" | 776 |
| 28 | limit 27 to english language | 762 |

Table 3.2b: Costs (Direct and Indirect Costs) search terms (EMBASE), performed May 9, 2022

| **Embase Classic+Embase <1947 to 2022 May 06>** | | |
| --- | --- | --- |
| 1 | Inflammatory bowel disease/ | 45077 |
| 2 | exp Crohn disease/ | 105411 |
| 3 | exp ulcerative colitis/ | 89491 |
| 4 | proctitis/ | 7581 |
| 5 | ileitis/ | 7344 |
| 6 | pancolitis/ | 2209 |
| 7 | proctocolitis/ | 1353 |
| 8 | ("Inflammatory bowel disease*" or ulcerative colitis or Crohn* or IBD or ileocolitis or ileitis or pancolitis or proctitis).af. | 237168 |
| 9 | or/1-8 [Set 2: IBD] | 237978 |
| 10 | "cost"/ | 64273 |
| 11 | exp "health care cost"/ | 319893 |
| 12 | "cost of illness"/ | 20577 |
| 13 | ((direct or health$ or indirect or medication$ or therap$ or surg$ or caregiv$ or prescription$ or hospital$ or drug$) adj5 (cost$ or expen$ or spending or payment$)).ti,ab,kf. | 277232 |
| 14 | "out-of-pocket".ti,ab,kf. | 10252 |
| 15 | absenteeism/ | 20476 |
| 16 | presenteeism/ | 1886 |
| 17 | (absenteeism or presenteeism).ti,ab,kf. | 11577 |
| 18 | ((absent$ or miss$) adj5 (work or employment)).ti,ab,kf. | 7502 |
| 19 | (financial adj (impact or toxicity or distress or burden or hardship or effect or difficult$)).ti,ab,kf. | 18437 |
| 20 | (economic adj (burden or impact or implication$ or hardship$ or difficult$)).ti,ab,kf. | 43493 |
| 21 | friction cost.ti,ab,kf. | 186 |
| 22 | material hardship$.ti,ab,kf. | 224 |
| 23 | (societ$ adj cost$).ti,ab,kf. | 3548 |
| 24 | labo?r market.ti,ab,kf. | 5396 |
| 25 | ((loss or lost) adj3 productivity).ti,ab,kf. | 7213 |
| 26 | deprivation.ti,ab,kf. | 102541 |
| 27 | poverty.ti,ab,kf. | 36818 |
| 28 | bankrupt$.ti,ab,kf. | 994 |
| 29 | medical leave/ | 7968 |
| 30 | hospitalization cost/ | 8651 |
| 31 | or/10-30 [Set 2: Costs of Care] | 754689 |
| 32 | 9 and 31 | 6059 |
| 33 | limit 32 to yr="2018-current" | 2198 |
| 34 | limit 33 to english language | 2162 |
| 35 | limit 34 to conference abstract | 951 |
| 36 | 34 not 35 | 1211 |


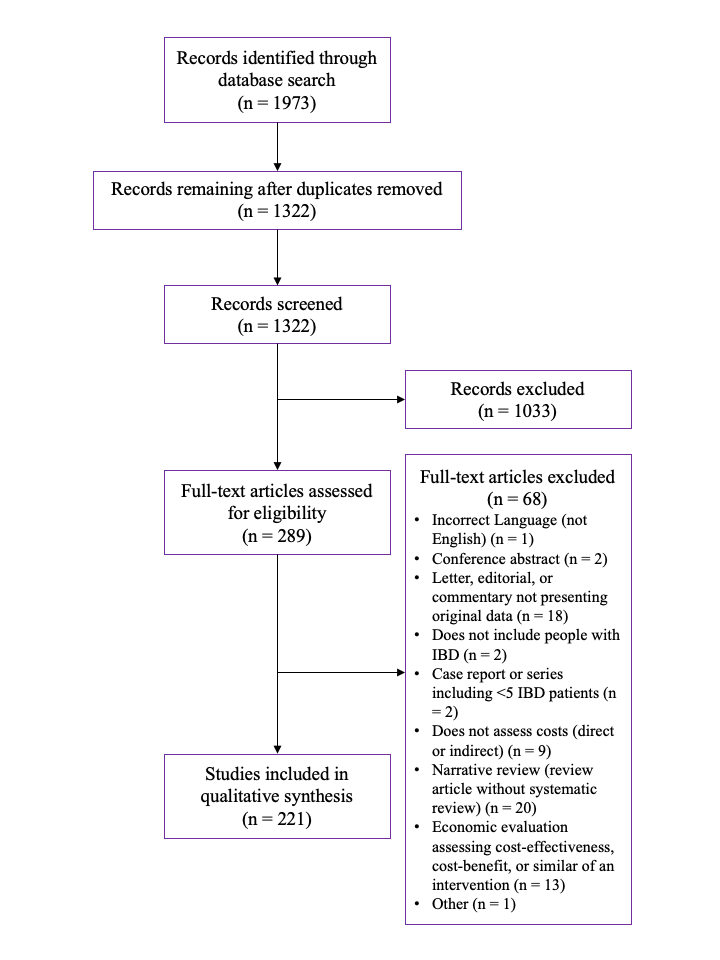


Figure 3: Flowchart detailing study selection for the Costs (Direct and Indirect Costs) systematic review

Table 4.1: Health Services Utilization inclusion and exclusion criteria

| **Inclusion Criteria** | **Exclusion Criteria** |
| --- | --- |
| ***Title and Abstract Review***   - Population-based observational study describing the frequency at which people with IBD, CD, or UC utilize any of the following types of health services:   - Hospitalizations   - Emergency department visits   - Outpatient visits   - Gastroenterologist visits   - Endoscopy   - Imaging (e.g., MRI, CT, ultrasound)   - Abdominal surgeries (e.g., intestinal resection, colectomy, pouch surgeries)   - Perianal surgeries; or - Population-based observational study comparing the frequency of health services utilization (see above list) among people with and without IBD, CD, or UC; or - Systematic review of studies summarizing the frequency at which people with IBD, CD, or UC utilize the types of health services listed above; or - Systematic review comparing the frequency of health services utilization (see above list) among people with and without IBD, CD, or UC; or - Observational study identifying factors associated with higher rates of any type of health services utilization (see above list). Studies may or may not be population-based and could also be secondary analysis of randomized controlled trials; or - Systematic review summarizing data on factors associated with higher rates of health services utilization (as outlined above).   *Note: Population-based studies are those that describe rates of health services utilization among all individuals living within a geographically well-defined political jurisdiction.* | ***Title and Abstract Review***   - Study is not in English - Conference abstract - Letter, editorial, or commentary not presenting original data - Case report or series including <5 IBD patients - Narrative review (review article without systematic review) - Does not include people with IBD - Does not describe rates of any of the outcomes listed in the inclusion criteria - Reports frequency of health services utilization but is not population-based - Compares the frequency of health services utilization in people with and without IBD but is not population-based |
| ***Full-Text Review***   - Population-based observational study describing the frequency at which people with IBD, CD, or UC utilize any of the following types of **IBD-associated** **or all-cause** health services:   - Hospitalizations   - Emergency department visits   - Outpatient visits   - Gastroenterologist visits   - Endoscopy   - Imaging (e.g., MRI, CT, ultrasound)   - Abdominal surgeries (e.g., intestinal resection, colectomy, pouch surgeries)   - Perianal surgeries; or - Population-based observational study comparing the frequency of health services utilization (see above list) among people with and without IBD, CD, or UC; or - Systematic review of studies summarizing the frequency at which people with IBD, CD, or UC utilize the types of health services listed above; or - Systematic review comparing the frequency of health services utilization (see above list) among people with and without IBD, CD, or UC; or - Observational study identifying **non-clinical (e.g., environmental factors, socioeconomic status, race/ethnicity)** factors associated with higher rates of any type of health services utilization (see above list). Studies may or may not be population-based and could also be secondary analysis of randomized controlled trials; or - Systematic review summarizing data on factors associated with higher rates of health services utilization (as outlined above).   *Note: Population-based studies are those that describe rates of health services utilization among all individuals living within a geographically well-defined political jurisdiction.*  *Note: IBD-associated or all-cause health services do not include those for other chronic conditions (including malignancy), infection, or cancer screening.*  *Note: Do not include studies describing the association between clinical factors (e.g., medication, disease phenotype, disease behaviour, serological markers) and health services utilization.* | ***Full-Text Review***   - Study is not in English - Conference abstract - Letter, editorial, or commentary not presenting original data - Case report or series including <5 IBD patients - Narrative review (review article without systematic review) - Does not include people with IBD - Does not describe rates of any of the outcomes listed in the inclusion criteria - Reports frequency of health services utilization but is not population-based - Compares the frequency of health services utilization in people with and without IBD but is not population-based - Evaluates the impact of a clinical factor (e.g., medication, disease phenotype/behaviour, serological marker) on IBD-associated health services utilization |

Table 4.2a: Health Services Utilization search terms (MEDLINE), performed May 9, 2022

| **Ovid MEDLINE: Epub Ahead of Print, In-Process & Other Non-Indexed Citations, Ovid MEDLINE® Daily and Ovid MEDLINE® <1946-Present>** | | |
| --- | --- | --- |
| 1 | exp Inflammatory Bowel Diseases/ | 91323 |
| 2 | exp Colitis, Ulcerative/ | 38388 |
| 3 | exp Crohn Disease/ | 42487 |
| 4 | exp Proctitis/ | 3168 |
| 5 | exp Enteritis/ | 14163 |
| 6 | ("Inflammatory bowel disease$" or ulcerative colitis or Crohn$ or IBD or ileocolitis or ileitis or pancolitis or proctitis).af. | 131238 |
| 7 | or/1-6 [Set 1: IBD] | 145490 |
| 8 | Hospitalization/sn, td [Statistics & Numerical Data, Trends] | 41394 |
| 9 | Patient Readmission/sn, td [Statistics & Numerical Data, Trends] | 11726 |
| 10 | Emergency Service, Hospital/sn, td [Statistics & Numerical Data, Trends] | 23348 |
| 11 | Ambulatory Care/sn, td [Statistics & Numerical Data, Trends] | 7022 |
| 12 | "Referral and Consultation"/sn, td [Statistics & Numerical Data, Trends] | 13302 |
| 13 | diagnostic imaging/sn, td [Statistics & Numerical Data, Trends] | 3539 |
| 14 | ultrasonography/sn, td [Statistics & Numerical Data, Trends] | 2042 |
| 15 | Magnetic Resonance Imaging/sn, td [Statistics & Numerical Data, Trends] | 6719 |
| 16 | Tomography, X-Ray Computed/sn, td [Statistics & Numerical Data, Trends] | 5862 |
| 17 | exp proctectomy/sn, td [Statistics & Numerical Data, Trends] | 178 |
| 18 | exp colectomy/sn, td [Statistics & Numerical Data, Trends] | 963 |
| 19 | exp enterostomy/sn, td [Statistics & Numerical Data, Trends] | 355 |
| 20 | endoscopy, digestive system/sn, td [Statistics & Numerical Data, Trends] | 356 |
| 21 | exp endoscopy, gastrointestinal/sn, td [Statistics & Numerical Data, Trends] | 3659 |
| 22 | ((frequen$ or rate$ or need$) adj5 (emergency adj5 (room$ or department$))).ti,ab,kf. | 5701 |
| 23 | ((frequen$ or rate$ or need$) adj5 (outpatient$ adj5 (visit$ or encounter$))).ti,ab,kf. | 797 |
| 24 | ((frequen$ or rate$ or need$) adj5 (gastroenterolog$ adj3 (visit$ or encounter$))).ti,ab,kf. | 23 |
| 25 | ((frequen$ or rate$ or need$ or utili$) adj5 ((MR or magnetic resonance or CT or computerized tomography) adj enterography)).ti,ab,kf. | 35 |
| 26 | ((frequen$ or rate$ or need$ or utili$) adj5 ultrasound$).ti,ab,kf. | 23504 |
| 27 | ((risk$ or frequen$ or rate$ or need$) adj5 ((intestinal or ileoc?ecal or ileocolic or ileocolonic) adj resect$)).ti,ab,kf. | 297 |
| 28 | Colonic Pouches/sn, td [Statistics & Numerical Data, Trends] | 48 |
| 29 | (((risk$ or frequen$ or rate$ or need$) adj5 (hospital adj3 (admission$ or admit$))) or hospitalization$ or (hospital adj3 (re?admit$ or re?admission$))).ti,ab,kf. | 191289 |
| 30 | Delayed Diagnosis/ | 7856 |
| 31 | (diagnos$ adj5 (delay$ or lag$)).ti,ab,kf. | 39874 |
| 32 | or/8-31 [Set 3: Health services utilization] | 341907 |
| 33 | 7 and 32 | 4020 |
| 34 | limit 33 to yr="2018-current" | 1555 |
| 35 | limit 34 to english language | 1530 |

Table 4.2b: Health Services Utilization search terms (EMBASE), performed May 9, 2022

| **Embase Classic+Embase <1947 to 2022 May 06>** | | |
| --- | --- | --- |
| 1 | Inflammatory bowel disease/ | 45077 |
| 2 | exp Crohn disease/ | 105411 |
| 3 | exp ulcerative colitis/ | 89491 |
| 4 | proctitis/ | 7581 |
| 5 | ileitis/ | 7344 |
| 6 | pancolitis/ | 2209 |
| 7 | proctocolitis/ | 1353 |
| 8 | ("Inflammatory bowel disease*" or ulcerative colitis or Crohn* or IBD or ileocolitis or ileitis or pancolitis or proctitis).af. | 237168 |
| 9 | or/1-8 [Set 1: IBD] | 237978 |
| 10 | (hospital management/ or hospital admission/ or hospital readmission/ or hospital utilization/ or hospitalization/) and (risk$ or frequen$ or rate$ or need$).ti,ab,kf. | 431177 |
| 11 | emergency ward/ and (frequen$ or rate$ or need$).ti,ab,kf. | 77202 |
| 12 | ambulatory care/ and (frequen$ or rate$ or need$).ti,ab,kf. | 14176 |
| 13 | patient referral/ | 138795 |
| 14 | diagnostic imaging/ and (frequen$ or rate$ or need$ or utili$).ti,ab,kf. | 55907 |
| 15 | (ultrasound/ or "point of care ultrasound"/) and (frequen$ or rate$ or need$ or utili$).ti,ab,kf. | 84154 |
| 16 | (nuclear magnetic resonance imaging/ or magnetic resonance enterography/) and (frequen$ or rate$ or need$ or utili$).ti,ab,kf. | 237260 |
| 17 | (computer assisted tomography/ or computed tomography enterography/) and (frequen$ or rate$ or need$ or utili$).ti,ab,kf. | 195238 |
| 18 | exp intestine endoscopy/ and (frequen$ or rate$ or need$ or utili$).ti,ab,kf. | 43235 |
| 19 | (exp colon surgery/ or exp colorectal surgery/ or exp enterostomy/ or ileum pouch/ or exp intestine anastomosis/ or exp intestine resection/ or exp rectum surgery/) and (risk$ or frequen$ or rate$ or need$).ti,ab,kf. | 88128 |
| 20 | ostomy/ and (risk$ or frequen$ or rate$ or need$).ti,ab,kf. | 850 |
| 21 | delayed diagnosis/ | 15779 |
| 22 | ((frequen$ or rate$ or need$) adj5 (emergency adj5 (room$ or department$))).ti,ab,kf. | 8669 |
| 23 | ((frequen$ or rate$ or need$) adj5 (outpatient$ adj5 (visit$ or encounter$))).ti,ab,kf. | 1344 |
| 24 | ((frequen$ or rate$ or need$) adj5 (gastroenterolog$ adj3 (visit$ or encounter$))).ti,ab,kf. | 53 |
| 25 | ((frequen$ or rate$ or need$ or utili$) adj5 ((MR or magnetic resonance or CT or computerized tomography) adj enterography)).ti,ab,kf. | 63 |
| 26 | ((frequen$ or rate$ or need$ or utili$) adj5 ultrasound$).ti,ab,kf. | 36870 |
| 27 | ((risk$ or frequen$ or rate$ or need$) adj5 ((intestinal or ileoc?ecal or ileocolic or ileocolonic) adj resect$)).ti,ab,kf. | 591 |
| 28 | (((risk$ or frequen$ or rate$ or need$) adj5 (hospital adj3 (admission$ or admit$))) or hospitalization$ or (hospital adj3 (re?admit$ or re?admission$))).ti,ab,kf. | 327274 |
| 29 | (diagnos$ adj5 (delay$ or lag$)).ti,ab,kf. | 63154 |
| 30 | or/10-29 [Set 2: Health services utilization] | 1408891 |
| 31 | 9 and 30 | 33385 |
| 32 | limit 31 to yr="2018-current" | 11892 |
| 33 | limit 32 to english language | 11747 |
| 34 | limit 33 to conference abstract | 6225 |
| 35 | 33 not 34 | 5522 |


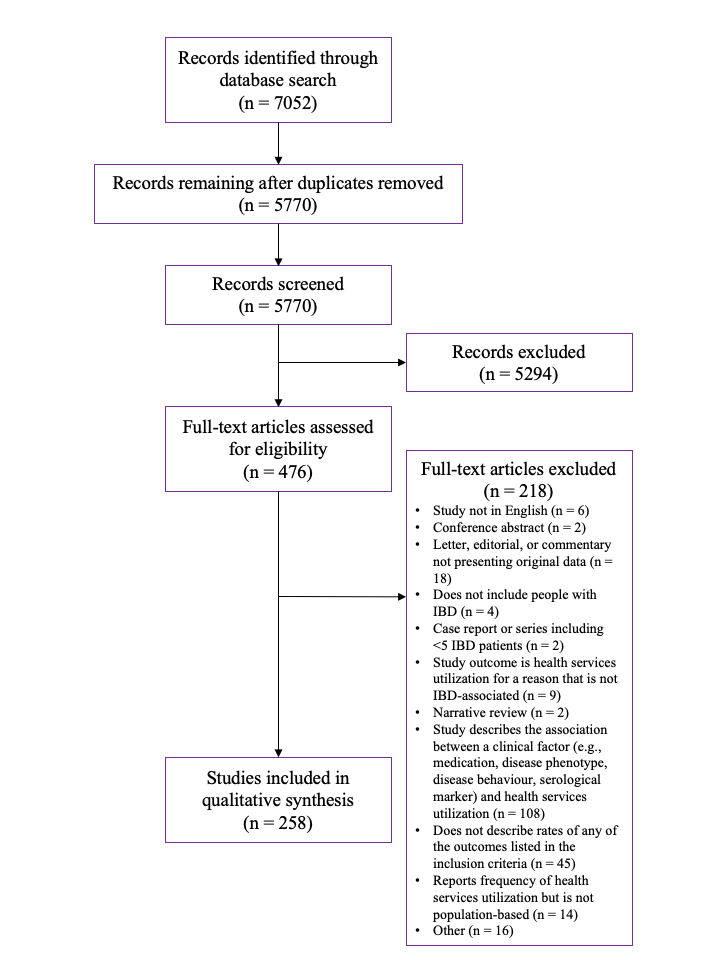


Figure 4: Flowchart detailing study selection for the Health Services Utilization systematic review

Table 5.1: COVID-19 inclusion and exclusion criteria

| **Inclusion Criteria** | **Exclusion Criteria** |
| --- | --- |
| - Randomized controlled trial, observational study, or systematic review; and - Study describes the impact of one of the following aspects on the COVID-19 pandemic on IBD patients:   - Epidemiology of COVID-19, including incidence/prevalence of infection, long COVID, or other post-viral illness   - Risk factors (including medication) for developing COVID-19   - Risk factors (including medication) for developing severe COVID-19, including risk of hospitalization, ICU admission, death, and long COVID or other post-viral illness   - Mental health   - Vaccine efficacy/effectiveness or safety, including risk of symptomatic infection, serious infection, and antibody response   - Anti-viral therapy; or - Study compares one of the following aspects of the COVID-19 pandemic on people with and without IBD:   - Epidemiology of COVID-19, including incidence/prevalence of infection, long COVID, or other post-viral illness   - Risk factors (including medication) for developing COVID-19   - Risk factors (including medication) for developing severe COVID-19, including risk of hospitalization, ICU admission, death, and long COVID or other post-viral illness   - Mental health   - Vaccine efficacy/effectiveness or safety, including risk of symptomatic infection, serious infection, and antibody response   - Anti-viral therapy - Qualitative study describing the experiences of IBD patients during the pandemic, including but not limited to:   - Barriers in accessing healthcare for their IBD   - Experiences with virtual care or changing models of care   *Note: Include studies where people with IBD are included in studies involving people with other chronic health conditions* | - Conference abstract - Study not in English - Case reports or series including <5 IBD patients - Commentary, editorial, or letter to the editor that does not provide novel data - Review article that did not include a systematic review - Basic or translational science study - Study describes the impact of COVID-19 on gastrointestinal system but is not specific to IBD - Study does not include patients with IBD - Study does not assess any of the listed outcomes |

Table 5.2a: COVID-19 search terms (medRxiv), performed April 28, 2022

| **medRxiv** | |
| --- | --- |
| 1 | "("inflammatory bowel disease" OR crohn OR crohn's OR "ulcerative colitis") AND (COVID OR SARS OR nCoV OR coronavirus)" and posted between "01 Jan, 2021 and 28 Apr, 2022" |

Table 5.2b: COVID-19 search terms (MEDLINE), performed April 28, 2022

| **Ovid MEDLINE: Epub Ahead of Print, In-Process & Other Non-Indexed Citations, Ovid MEDLINE® Daily and Ovid MEDLINE® <1946-Present>** | | |
| --- | --- | --- |
| 1 | exp Coronavirus/ | 135926 |
| 2 | exp Coronavirus Infections/ | 167469 |
| 3 | exp coronaviridae/ | 137123 |
| 4 | exp coronaviridae infections/ | 168388 |
| 5 | (2019 nCoV or 2019nCoV or 2019-novel CoV).ti,ab,kw. | 1874 |
| 6 | COVID.mp. | 241494 |
| 7 | (COVID19 or "COVID-19" or "COVID-19").ti,ab,kw. | 218419 |
| 8 | (nCov 2019 or nCov 19).ti,ab,kw. | 696 |
| 9 | ("SARS-CoV-2" or "SARS-CoV2" or SARSCoV2 or "SARSCoV-2" or SARS2).af. | 159993 |
| 10 | ("SARS coronavirus 2" or "SARS-like coronavirus" or "Severe Acute Respiratory Syndrome Coronavirus-2" or "Severe Acute Respiratory Syndrome Coronavirus 2").af. | 24597 |
| 11 | (("severe acute respiratory" or pneumonia*) adj5 (coronavir* or COVID*)).af. | 69560 |
| 12 | (corona vir* or coronoavir* or neocorona vir* or neocoronavir*).ti,ab,kw. | 2991 |
| 13 | ((wuhan or hubei or Hunan) and (severe acute respiratory or pneumonia*) and outbreak*).af. | 1958 |
| 14 | ((wuhan or hubei or Hunan) and coronavir*).af. | 8720 |
| 15 | or/1-14 [Set 1: SARS-CoV-2] | 266811 |
| 16 | exp Inflammatory Bowel Diseases/ | 91073 |
| 17 | exp Colitis, Ulcerative/ | 38287 |
| 18 | exp Crohn Disease/ | 42409 |
| 19 | exp Proctitis/ | 3165 |
| 20 | exp Enteritis/ | 14132 |
| 21 | ("Inflammatory bowel disease$" or ulcerative colitis or Crohn$ or IBD or ileocolitis or ileitis or pancolitis or proctitis).af. | 130915 |
| 22 | or/16-21 [Set 2: IBD] | 145137 |
| 23 | 15 and 22 | 1186 |
| 24 | limit 23 to yr="2021-Current" | 728 |
| 25 | limit 24 to english language | 714 |

Table 5.2c: COVID-19 search terms (EMBASE), performed April 28, 2022

| **Embase Classic+Embase <1947 to 2022 April 27>** | | |
| --- | --- | --- |
| 1 | exp Coronavirus infection/ | 230498 |
| 2 | exp Coronavirinae/ | 84906 |
| 3 | exp coronaviridae/ | 85909 |
| 4 | exp coronaviridae infection/ | 230673 |
| 5 | (2019 nCoV or 2019nCoV or 2019-novel CoV).ti,ab,kw. | 1977 |
| 6 | COVID.af. | 241205 |
| 7 | (COVID19 or "COVID-19" or "COVID-19").ti,ab,kw. | 231964 |
| 8 | (nCov 2019 or nCov 19).ti,ab,kw. | 752 |
| 9 | ("SARS-CoV-2" or "SARS-CoV2" or SARSCoV2 or "SARSCoV-2" or SARS2).af. | 98005 |
| 10 | ("SARS coronavirus 2" or "SARS-like coronavirus" or "Severe Acute Respiratory Syndrome Coronavirus-2" or "Severe Acute Respiratory Syndrome Coronavirus 2").af. | 74177 |
| 11 | (("severe acute respiratory" or pneumonia*) adj5 (coronavir* or COVID*)).af. | 87259 |
| 12 | (corona vir* or coronavir* or neocorona vir* or neocoronavir*).ti,ab,kw. | 102596 |
| 13 | ((wuhan or Hubei or Hunan) and (severe acute respiratory or pneumonia*) and outbreak*).mp. | 1759 |
| 14 | ((wuhan or hubei or Hunan) and coronavir*).mp. | 7603 |
| 15 | or/1-14 [Set 1: SARS-CoV-2] | 305422 |
| 16 | Inflammatory bowel disease/ | 44887 |
| 17 | exp Crohn disease/ | 105320 |
| 18 | exp ulcerative colitis/ | 89402 |
| 19 | proctitis/ | 7576 |
| 20 | ileitis/ | 7334 |
| 21 | pancolitis/ | 2208 |
| 22 | proctocolitis/ | 1348 |
| 23 | ("Inflammatory bowel disease$" or ulcerative colitis or Crohn$ or IBD or ileocolitis or ileitis or pancolitis or proctitis).af. | 236821 |
| 24 | or/16-23 [Set 2: IBD] | 237627 |
| 25 | 15 and 24 | 2114 |
| 26 | limit 25 to yr="2021-Current" | 1467 |
| 27 | limit 26 to english language | 1453 |

Table 5.2d: COVID-19 search terms (PsycINFO), performed April 28, 2022

| **APA PsycInfo <1806 to April Week 4 2022>** | | |
| --- | --- | --- |
| 1 | exp coronavirus/ | 10966 |
| 2 | (2019 nCoV or 2019nCoV or 2019-novel CoV).af. | 1084 |
| 3 | COVID.af. | 20774 |
| 4 | (COVID19 or "COVID-19" or "COVID 19").af. | 20687 |
| 5 | (nCov 2019 or nCov 19).af. | 42 |
| 6 | ("SARS-CoV-2" or "SARS-CoV2" or SARSCoV2 or "SARSCoV-2" or SARS2).af. | 4609 |
| 7 | ("SARS coronavirus 2" or "SARS-like coronavirus" or "Severe Acute Respiratory Syndrome Coronavirus-2" or "Severe Acute Respiratory Syndrome Coronavirus 2").af. | 622 |
| 8 | (("severe acute respiratory" or pneumonia*) adj5 (coronavir* or COVID*)).af. | 2897 |
| 9 | (corona vir* or coronavir* or neocorona vir* or neocoronavir*).af. | 11992 |
| 10 | ((wuhan or Hubei or Hunan) and (severe acute respiratory or pneumonia*) and outbreak*).af. | 1037 |
| 11 | ((wuhan or hubei or Hunan) and coronavir*).af. | 2060 |
| 12 | or/1-11 [Set 1: SARS-CoV-2] | 22017 |
| 13 | exp colon disorders/ | 4649 |
| 14 | exp ulcerative colitis/ or exp colitis/ | 489 |
| 15 | ("Inflammatory bowel disease*" or ulcerative colitis or Crohn* or IBD or ileocolitis or ileitis or pancolitis or proctitis).af. | 10075 |
| 16 | or/13-15 | 13324 |
| 17 | 12 and 16 | 115 |
| 18 | limit 17 to yr="2021-Current" | 79 |
| 19 | limit 18 to english language | 59 |

Table 5.2e: COVID-19 search terms (Cochrane Library), performed April 28, 2022

| **Cochrane Library (Wiley)** | | |
| --- | --- | --- |
| #1 | MeSH descriptor: [SARS-CoV-2] explode all trees | 901 |
| #2 | MeSH descriptor: [Coronavirus] explode all trees | 914 |
| #3 | MeSH descriptor: [Severe Acute Respiratory Syndrome] this term only | 371 |
| #4 | MeSH descriptor: [COVID-19] this term only | 1550 |
| #5 | MeSH descriptor: [Coronaviridae] explode all trees | 916 |
| #6 | MeSH descriptor: [Coronavirus Infections] this term only | 682 |
| #7 | ("2019 nCoV"):ti,ab,kw OR ("2019nCoV"):ti,ab,kw OR ("2019-novel | 11 |
| #8 | (COVID):ti,ab,kw OR (COVID19):ti,ab,kw OR ("COVID-19"):ti,ab,kw | 10077 |
| #9 | ("nCov 2019"):ti,ab,kw OR ("nCov 19"):ti,ab,kw | 72 |
| #10 | ("SARS-CoV-2"):ti,ab,kw OR ("SARS-CoV2"):ti,ab,kw OR (SARSCoV2):ti,ab,kw OR ("SARSCoV-2"):ti,ab,kw OR (SARS2):ti,ab,kw | 4015 |
| #11 | ("SARS coronavirus 2"):ti,ab,kw OR ("SARS-like coronavirus"):ti,ab,kw OR ("Severe acute respiratory syndrome coronavirus-2"):ti,ab,kw OR ("Severe acute respiratory syndrome coronavirus 2"):ti,ab,kw | 991 |
| #12 | (("severe acute respiratory"):ti,ab,kw OR (pneumonia*):ti,ab,kw) AND ((coronavir*):ti,ab,kw OR (COVID*):ti,ab,kw) | 2952 |
| #13 | ("corona vir*"):ti,ab,kw OR (coronavir*):ti,ab,kw OR ("neocorona vir*"):ti,ab,kw OR (neocoronavir*):ti,ab,kw | 5936 |
| #14 | ((wuhan):ti,ab,kw OR (Hubei):ti,ab,kw OR (Hunan):ti,ab,kw) AND (("severe acute respiratory"):ti,ab,kw OR (pneumonia*):ti,ab,kw) AND (outbreak*):ti,ab,kw | 53 |
| #15 | ((wuhan):ti,ab,kw OR (Hubei):ti,ab,kw OR (Hunan):ti,ab,kw) AND ((coronavir*):ti,ab,kw OR (COVID*):ti,ab,kw) | 256 |
| #16 | {OR #1-#15} | 10666 |
| #17 | MeSH descriptor: [Inflammatory Bowel Diseases] explode all trees | 3681 |
| #18 | MeSH descriptor: [Crohn Disease] explode all trees | 1736 |
| #19 | MeSH descriptor: [Colitis, Ulcerative] explode all trees | 1747 |
| #20 | MeSH descriptor: [Proctitis] this term only | 133 |
| #21 | MeSH descriptor: [Ileitis] explode all trees | 64 |
| #22 | MeSH descriptor: [Proctocolitis] this term only | 29 |
| #23 | ("inflammatory bowel disease*"):ti,ab,kw OR ("ulcerative colitis"):ti,ab,kw OR (Crohn*):ti,ab,kw OR (IBD):ti,ab,kw | 10935 |
| #24 | (ileocolitis):ti,ab,kw OR (ileitis):ti,ab,kw OR (pancolitis):ti,ab,kw OR | 820 |
| #25 | {OR #17-#24} | 11534 |
| #26 | #16 AND #25 | 32 |

Table 5.2f: COVID-19 search terms (CINAHL), performed April 28, 2022

| **CINAHL (EBSCO)** | | |
| --- | --- | --- |
| S1 | (MH "Coronavirus+") OR (MH "Coronavirus  Infections+") OR (MH "COVID-19") | Search modes - Boolean/Phrase |
| S2 | TX (2019 nCoV or 2019nCoV or 2019-novel  CoV) | Search modes - Boolean/Phrase |
| S3 | TX COVID | Search modes - Boolean/Phrase |
| S4 | TX (COVID19 or "COVID- 19" or "COVID 19") | Search modes - Boolean/Phrase |
| S5 | TX nCov 2019 or nCov 19 | Search modes - Boolean/Phrase |
| S6 | TX ("SARS-CoV-2" or "SARS-CoV2" or  SARSCoV2 or "SARSCoV-2" or SARS2) | Search modes - Boolean/Phrase |
| S7 | TX ("SARS coronavirus 2" or "SARS-like  coronavirus" or "Severe Acute Respiratory Syndrome Coronavirus-2" or "Severe Acute Respiratory Syndrome Coronavirus 2") | Search modes - Boolean/Phrase |
| S8 | TX ( "severe acute respiratory" OR  pneumonia* ) AND TX ( coronavir* OR COVID* ) | Search modes - Boolean/Phrase |
| S9 | TX (corona vir* or coronavir* or neocorona  vir* or neocoronavir*) | Search modes - Boolean/Phrase |
| S10 | TX ((wuhan or Hubei or Hunan) and (severe acute  respiratory or pneumonia*) and outbreak*) | Search modes - Boolean/Phrase |
| S11 | TX ((wuhan or hubei or Hunan) and coronavir*) | Search modes - Boolean/Phrase |
| S12 | S1 OR S2 OR S3 OR S4 OR S5 OR S6 OR S7 OR S8 OR S9 OR S10 OR S11 | Search modes - Boolean/Phrase |
| S13 | (MH "Inflammatory Bowel Diseases+") | Search modes - Boolean/Phrase |
| S14 | (MH “Crohn Disease”) | Search modes - Boolean/Phrase |
| S15 | (MH "Colitis, Ulcerative") | Search modes - Boolean/Phrase |
| S16 | (MH "Ileitis+") | Search modes - Boolean/Phrase |
| S17 | TX ("Inflammatory bowel disease*" or ulcerative  colitis or Crohn* or IBD or ileocolitis or ileitis or pancolitis or proctitis) | Search modes - Boolean/Phrase |
| S18 | S13 OR S14 OR S15 OR S16 OR S17 | Search modes - Boolean/Phrase |
| S19 | S12 AND S18 | Search modes - Boolean/Phrase |
| S20 | S12 AND S18 | Limiters - Published Date: 20210101-20220531; English Language Search modes - Boolean/Phrase |


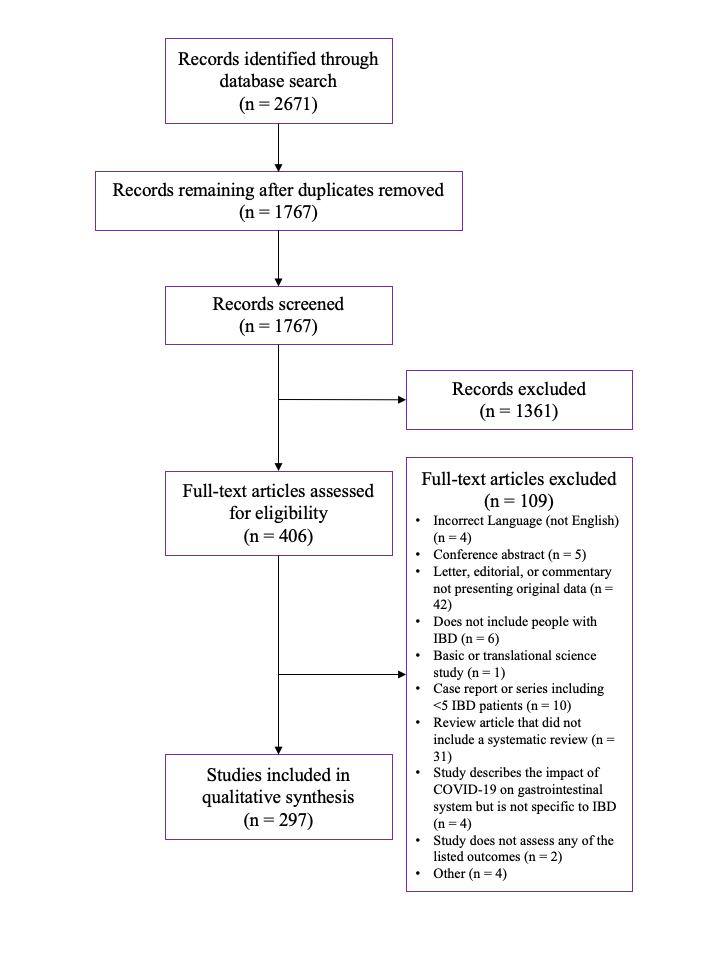


Figure 5: Flowchart detailing study selection for the COVID-19 systematic review

Table 6.1: Mental Health inclusion and exclusion criteria

| **Inclusion Criteria** | **Exclusion Criteria** |
| --- | --- |
| Part 1: Epidemiology   - Population-based observational study describing the incidence or prevalence of clinician-diagnosed anxiety, depression, or other mental health conditions in people with inflammatory bowel disease (IBD), Crohn’s disease (CD), or ulcerative colitis (UC) - Population-based observational study comparing the frequency of clinician-diagnosed anxiety, depression, or other mental health concerns in people with and without IBD, CD, or UC - Systematic review of the incidence or prevalence of mental health conditions in people with IBD/CD/UC or comparing incidence or prevalence of mental health conditions in people with and without IBD/CD/UC   *Note: Clinician-diagnosed mental health conditions can include studies that use healthcare encounters (outpatient, emergency department, or hospitalizations) with a diagnostic code for mental health concerns to identify individuals with anxiety, depression, or other mental health concerns*  *Note: Population-based studies are those that provide estimates of incidence or prevalence of mental health concerns among all IBD patients living within a geographically well-defined political jurisdiction.*  Part 2: Symptoms of anxiety and depression and the role of stress and quality of life   - Observational studies describing the frequency of anxiety, depressive, or related symptoms using validated indices among people with IBD, CD, or UC; or - Observational studies comparing the frequency of anxiety, depressive, or related symptoms using validated indices among people with and without IBD, CD, or UC; or - Observational studies describing the role of stress in the psychosocial wellbeing of people with IBD, CD, or UC; or - Observational studies describing the role of stress in symptoms of IBD or other IBD outcomes (e.g., hospitalization, surgery); or - Observational studies describing the interplay between mental health and quality of life in people with IBD, CD, or UC; or - Systematic review on any of the above topics.   Part 3: Improving mental health in people with IBD   - Randomized controlled trials evaluating an intervention aimed to improve some aspect of mental health or psychosocial well-being in patients with IBD, CD, or UC; or - Systematic review of randomized controlled trials evaluating intervention(s) to improve mental health or psychosocial well-being in patients with IBD, CD, or UC. | - Conference abstract - Study not in English - Case reports or series including <5 IBD patients - Commentary, editorial, or letter to the editor that does not provide novel data - Review article that did not include a systematic review - Basic or translational science study - Qualitative study - Study does not include patients with IBD - Study describes incidence or prevalence of physician-diagnosed mental health condition but is not population-based - Study describes frequency of mental health symptoms in people with IBD but does not use a validated scale to measure symptoms - Study evaluates the impact of an intervention but is not a randomized controlled trial |

Table 6.2a: Mental Health search terms (MEDLINE), performed May 5, 2022

| **Ovid MEDLINE: Epub Ahead of Print, In-Process & Other Non-Indexed Citations, Ovid MEDLINE® Daily and Ovid MEDLINE® <1946-Present>** | | |
| --- | --- | --- |
| 1 | exp Inflammatory Bowel Diseases/ | 91261 |
| 2 | exp Colitis, Ulcerative/ | 38371 |
| 3 | exp Crohn Disease/ | 42469 |
| 4 | exp Proctitis/ | 3166 |
| 5 | exp Enteritis/ | 14155 |
| 6 | ("Inflammatory bowel disease$" or ulcerative colitis or Crohn$ or IBD or ileocolitis or ileitis or pancolitis or proctitis).mp. | 129011 |
| 7 | or/1-6 [Set 1: IBD] | 143405 |
| 8 | exp anxiety disorders/ or mood disorders/ or depressive disorder/ | 160818 |
| 9 | Anxiety/ | 98023 |
| 10 | depression/ or exp stress, psychological/ | 272584 |
| 11 | depress$.ti,ab,kf. | 524634 |
| 12 | (anxiet$ or anxious$).ti,ab,kf. | 244191 |
| 13 | stress$.ti,ab,kf. | 991201 |
| 14 | suicid$.ti,ab,kf. | 89239 |
| 15 | Resilience, Psychological/ | 7826 |
| 16 | resilien$.ti,ab,kf. | 48119 |
| 17 | exp Adaptation, Psychological/ | 136216 |
| 18 | (coping adj3 (strateg$ or mechanism$)).ti,ab,kf. | 21595 |
| 19 | exp Self-Injurious Behavior/ | 79301 |
| 20 | (self adj3 harm$).ti,ab,kf. | 8554 |
| 21 | or/8-20 [Set 2: Mental Health] | 1880871 |
| 22 | 7 and 21 | 5911 |
| 23 | limit 22 to yr="2018-Current" | 2080 |
| 24 | limit 23 to english language | 2046 |
| 25 | 24 not ((exp animal/ or exp invertebrate/ or animal experiment/ or animal model/ or exp plant/ or exp fungus/) not exp human/) | 1686 |

Table 6.2b: Mental Health search terms (EMBASE), performed May 5, 2022

| **Embase Classic+Embase <1947 to 2022 May 04>** | | |
| --- | --- | --- |
| 1 | Inflammatory bowel disease/ | 45039 |
| 2 | exp Crohn disease/ | 105402 |
| 3 | exp ulcerative colitis/ | 89478 |
| 4 | proctitis/ | 7581 |
| 5 | ileitis/ | 7341 |
| 6 | pancolitis/ | 2209 |
| 7 | proctocolitis/ | 1352 |
| 8 | ("Inflammatory bowel disease*" or ulcerative colitis or Crohn* or IBD or ileocolitis or ileitis or pancolitis or proctitis).af. | 237110 |
| 9 | or/1-8 [Set 2: IBD] | 237919 |
| 10 | exp anxiety disorder/ | 288447 |
| 11 | mood disorder/ | 48536 |
| 12 | exp depression/ | 557754 |
| 13 | anxiety/ | 256662 |
| 14 | exp anxiety assessment/ | 56265 |
| 15 | exp physiological stress/ | 301901 |
| 16 | psychological resilience/ | 7108 |
| 17 | exp coping behavior/ | 76255 |
| 18 | psychological adjustment/ | 1685 |
| 19 | automutilation/ | 22057 |
| 20 | exp suicidal behavior/ | 121198 |
| 21 | depress$.ti,ab,kf. | 740496 |
| 22 | (anxiet$ or anxious$).ti,ab,kf. | 354205 |
| 23 | stress$.ti,ab,kf. | 1295597 |
| 24 | suicid$.ti,ab,kf. | 116671 |
| 25 | resilien$.ti,ab,kf. | 54367 |
| 26 | (coping adj3 (strateg$ or mechanism$)).ti,ab,kf. | 27939 |
| 27 | (self adj3 harm$).ti,ab,kf. | 11168 |
| 28 | or/10-27 [Set 2: Mental Health] | 2628085 |
| 29 | 9 and 28 | 13042 |
| 30 | limit 29 to yr="2018-current" | 4935 |
| 31 | limit 30 to english language | 4864 |
| 32 | 31 not ((exp animal/ or exp invertebrate/ or nonhuman/ or animal experiment/ or animal tissue/ or animal model/ or exp plant/ or exp fungus/) not (exp human/ or human tissue/)) | 4103 |

Table 6.2c: Mental Health search terms (PsycINFO), performed May 5, 2022

| **APA PsycInfo <1806 to April Week 4 2022>** | | |
| --- | --- | --- |
| 1 | exp ulcerative colitis/ or exp colitis/ | 489 |
| 2 | ("Inflammatory bowel disease*" or ulcerative colitis or Crohn* or IBD or ileocolitis or ileitis or pancolitis or proctitis).af. | 10075 |
| 3 | exp colon disorders/ | 4649 |
| 4 | or/1-3 [Set 1: IBD] | 13324 |
| 5 | anxiety/ | 70149 |
| 6 | anxiety disorders/ | 19920 |
| 7 | affective disorders/ | 15214 |
| 8 | major depression/ | 138307 |
| 9 | "depression (emotion)"/ | 26517 |
| 10 | stress/ or perceived stress/ or stress management/ | 74884 |
| 11 | "resilience (psychological)"/ | 17965 |
| 12 | helplessness/ or hopelessness/ or self-efficacy/ | 28833 |
| 13 | exp coping behavior/ | 51600 |
| 14 | exp self-injurious behavior/ | 7037 |
| 15 | exp suicide/ | 38145 |
| 16 | suicidal ideation/ or attempted suicide/ or suicidality/ | 21012 |
| 17 | depress$.ti,ab,hw,id. | 340077 |
| 18 | stress$.ti,ab,hw,id. | 308324 |
| 19 | suicid$.ti,ab,hw,id. | 72180 |
| 20 | resilien$.ti,ab,hw,id. | 39663 |
| 21 | (coping adj3 (strateg$ or mechanism$)).ti,ab,hw,id. | 28260 |
| 22 | (self adj3 harm$).ti,ab,hw,id. | 7978 |
| 23 | or/5-22 [Set 2: Mental Health] | 761035 |
| 24 | 4 and 23 | 3839 |
| 25 | limit 24 to yr="2018-current" | 1186 |
| 26 | limit 25 to english language | 1091 |
| 27 | limit 26 to human | 948 |


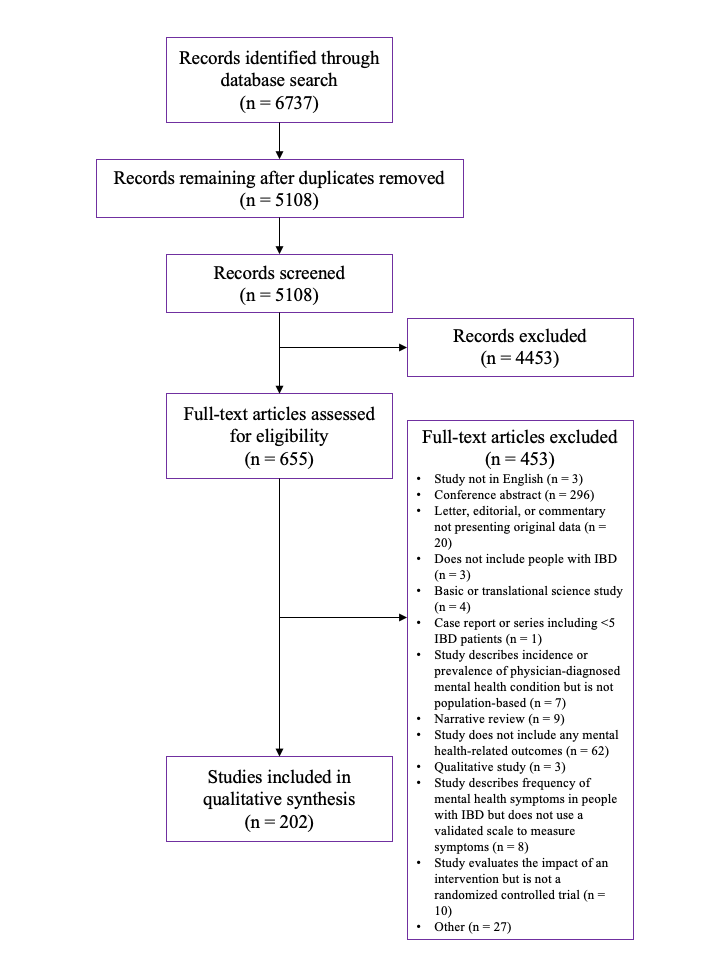


Figure 6: Flowchart detailing study selection for the Mental Health systematic review

Table 7.1: Pediatric Growth, Development, and Education inclusion and exclusion criteria

| **Inclusion Criteria** | **Exclusion Criteria** |
| --- | --- |
| - Observational study describing growth in children with inflammatory bowel disease (IBD), Crohn’s disease (CD), or ulcerative colitis UC); or - Observational study comparing growth in children with and without IBD, CD, or UC; or - Observational study or randomized controlled trial evaluating the impact of IBD therapy on growth in children with IBD, CD, or UC; or - Observational study describing puberty or other developmental outcomes in children with IBD, CD, or UC; or - Observational study comparing puberty or other developmental outcomes in children with and without IBD, CD, or UC; or - Observational study or randomized controlled trial evaluating the impact of IBD therapy on puberty or other developmental outcomes; or - Observational or qualitative study describing educational achievement, aspirations, barriers to success, or other education-related outcomes in children or youth with IBD in an observational or qualitative study - Observational study comparing educational achievement or aspirations in children with and without IBD; or - Systematic review on any of the above topics   *Note: Include studies where youth with IBD are included in studies involving youth with other chronic health conditions*  *Note: Observational studies include cohort, case-control, or cross-sectional studies* | - Does not include children with inflammatory bowel disease - Case series or report involving <5 IBD patients - Letter, commentary, or editorial that does not include original data - Narrative review article - Conference abstract - Study is not written in English - Does not assess any of the listed outcomes - Other |

Table 7.2a: Pediatric Growth, Development, and Education search terms (MEDLINE), performed May 4, 2022

| **Ovid MEDLINE: Epub Ahead of Print, In-Process & Other Non-Indexed Citations, Ovid MEDLINE® Daily and Ovid MEDLINE® <1946-Present>** | | |
| --- | --- | --- |
| 1 | exp Inflammatory Bowel Diseases/ | 91310 |
| 2 | exp Colitis, Ulcerative/ | 38390 |
| 3 | exp Crohn Disease/ | 42484 |
| 4 | exp Proctitis/ | 3165 |
| 5 | exp Enteritis/ | 14153 |
| 6 | ("Inflammatory bowel disease$" or ulcerative colitis or Crohn$ or IBD or ileocolitis or ileitis or pancolitis or proctitis).af. | 131202 |
| 7 | or/1-6 [Set 1: IBD] | 145447 |
| 8 | sexual development/ or sexual maturation/ | 15084 |
| 9 | exp Puberty/ | 18680 |
| 10 | puberty, delayed/ or puberty, precocious/ | 6154 |
| 11 | thelarche.ti,ab,kf. | 569 |
| 12 | menarche.ti,ab,kf. | 9024 |
| 13 | adrenarche.ti,ab,kf. | 729 |
| 14 | pubert$.ti,ab,kf. | 41838 |
| 15 | (sex$ adj5 (matur$ or develop$)).ti,ab,kf. | 35142 |
| 16 | early matur$.af. | 1800 |
| 17 | first menstrua$.ti,ab,kf. | 379 |
| 18 | tanner stage.ti,ab,kf. | 1749 |
| 19 | pubarche.ti,ab,kf. | 521 |
| 20 | (breast$ adj3 develop$).ti,ab,kf. | 16204 |
| 21 | (genital$ adj3 stage$).ti,ab,kf. | 295 |
| 22 | first ejaculation$.ti,ab,kf. | 109 |
| 23 | or/8-22 [Set 2: Puberty] | 108852 |
| 24 | exp Body Size/ | 370363 |
| 25 | Body Mass Index/ | 143342 |
| 26 | "growth and development"/ or growth/ or human development/ | 31705 |
| 27 | grow$.ti,ab,kw. | 2053304 |
| 28 | (height or weight).ti,ab,kf. | 1030041 |
| 29 | Growth Disorders/ | 18194 |
| 30 | or/24-29 [Set 3: Growth] | 3160677 |
| 31 | education/ or schools/ | 67706 |
| 32 | Universities/ | 48466 |
| 33 | exp Educational Status/ | 57339 |
| 34 | exp Educational Measurement/ | 161417 |
| 35 | achievement/ or aspirations, psychological/ or goals/ | 34779 |
| 36 | Career Choice/ | 24923 |
| 37 | ((educat$ or academic$) adj5 (achiev$ or goal$ or ascertainment or aspir$)).ti,ab,kf. | 17467 |
| 38 | (career adj (goal$ or aspir$)).ti,ab,kf. | 972 |
| 39 | school achiev$.ti,ab,kw. | 1181 |
| 40 | absenteeism/ | 9623 |
| 41 | Presenteeism/ | 515 |
| 42 | (school$ adj5 (absen$ or miss$)).ti,ab,kf. | 4598 |
| 43 | or/31-42 [Set 3: Education] | 394981 |
| 44 | Pediatrics/ | 57464 |
| 45 | Adolescent/ | 2175759 |
| 46 | Young Adult/ | 991189 |
| 47 | Adolescent Medicine/ | 1559 |
| 48 | Adolescent Health Services/ | 5839 |
| 49 | child health services/ or early intervention, educational/ | 24506 |
| 50 | child/ or child, preschool/ or infant/ | 2333865 |
| 51 | p?ediatric$.af. | 1369029 |
| 52 | young adult?.af. | 1058806 |
| 53 | (teen$ or youth or adolescen$ or child$).af. | 4212798 |
| 54 | emerging adult?.af. | 2638 |
| 55 | (young m#n or young wom#n or young male? or young female?).ti,ab,kf. | 78558 |
| 56 | (juvenile or young person? or young people or young adult$).ti,ab,kf. | 223580 |
| 57 | child$.ti,ab,kf. | 1578566 |
| 58 | or/44-57 [Set 5: Children and Adolescents] | 5240640 |
| 59 | 7 and (23 or 30 or 43) and 58 | 3704 |
| 60 | limit 59 to yr="2018-Current" | 914 |
| 61 | limit 60 to english language | 892 |

Table 7.2b: Pediatric Growth, Development, and Education search terms (EMBASE), performed May 4, 2022

| **Embase Classic+Embase <1947 to 2022 May 03>** | | |
| --- | --- | --- |
| 1 | Inflammatory bowel disease/ | 45025 |
| 2 | exp Crohn disease/ | 105394 |
| 3 | exp ulcerative colitis/ | 89470 |
| 4 | proctitis/ | 7581 |
| 5 | ileitis/ | 7341 |
| 6 | pancolitis/ | 2209 |
| 7 | proctocolitis/ | 1352 |
| 8 | ("Inflammatory bowel disease*" or ulcerative colitis or Crohn* or IBD or ileocolitis or ileitis or pancolitis or proctitis).af. | 237084 |
| 9 | or/1-8 [Set 1: IBD] | 237893 |
| 10 | sexual development/ or sexual maturation/ or sexual maturity/ | 31392 |
| 11 | exp puberty/ | 50913 |
| 12 | delayed puberty/ | 3727 |
| 13 | precocious puberty/ | 9328 |
| 14 | breast development/ | 4634 |
| 15 | adrenarche/ | 953 |
| 16 | thelarche.ti,ab,kf. | 936 |
| 17 | menarche.ti,ab,kf. | 13219 |
| 18 | adrenarche.ti,ab,kf. | 1137 |
| 19 | pubert$.ti,ab,kf. | 61174 |
| 20 | (sex$ adj5 (matur$ or develop$)).ti,ab,kf. | 45371 |
| 21 | early matur$.af. | 1956 |
| 22 | first menstrua$.ti,ab,kf. | 610 |
| 23 | tanner stage.ti,ab,kf. | 3088 |
| 24 | pubarche.ti,ab,kf. | 862 |
| 25 | (breast$ adj3 develop$).ti,ab,kf. | 23036 |
| 26 | (genital$ adj3 stage$).ti,ab,kf. | 494 |
| 27 | first ejaculation$.ti,ab,kf. | 142 |
| 28 | or/10-27 [Set 2: Puberty] | 170207 |
| 29 | body size/ | 29406 |
| 30 | body height/ | 82865 |
| 31 | exp body weight/ | 619616 |
| 32 | body mass/ | 538468 |
| 33 | body growth/ | 7793 |
| 34 | growth disorder/ | 18400 |
| 35 | physical development/ | 4921 |
| 36 | "growth, development and aging"/ | 233184 |
| 37 | child growth/ | 14340 |
| 38 | grow$.ti,ab,kw. | 2516737 |
| 39 | (height or weight).ti,ab,kf. | 1437276 |
| 40 | or/29-39 [Set 3: Growth] | 4463049 |
| 41 | education/ or exp academic achievement/ or "outcome of education"/ or primary education/ or exp school attendance/ or secondary education/ | 520194 |
| 42 | absenteeism/ | 20476 |
| 43 | exp school/ | 409595 |
| 44 | exp student/ | 304569 |
| 45 | exp educational status/ | 113826 |
| 46 | achievement/ or achievement test/ | 42590 |
| 47 | motivation/ | 120969 |
| 48 | decision making/ | 262633 |
| 49 | ((educat$ or academic$) adj5 (achiev$ or goal$ or ascertainment or aspir$)).ti,ab,kf. | 23353 |
| 50 | (career adj (goal$ or aspir$)).ti,ab,kf. | 1267 |
| 51 | school achiev$.ti,ab,kw. | 1415 |
| 52 | presenteeism/ | 1886 |
| 53 | (school$ adj5 (absen$ or miss$)).ti,ab,kf. | 6517 |
| 54 | or/41-53 [Set 4: Education] | 1548589 |
| 55 | pediatrics/ | 94527 |
| 56 | adolescent/ | 1809401 |
| 57 | young adult/ | 456999 |
| 58 | adolescence/ | 47011 |
| 59 | infant/ or child/ or baby/ | 2593053 |
| 60 | child health/ | 32331 |
| 61 | adolescent health/ | 9802 |
| 62 | child health care/ or early childhood intervention/ | 42429 |
| 63 | preschool child/ | 669918 |
| 64 | (teen$ or youth or adolescen$).af. | 2071284 |
| 65 | p?ediatric$.af. | 2542273 |
| 66 | young adult?.af. | 552516 |
| 67 | emerging adult?.af. | 3010 |
| 68 | (young m#n or young wom#n or young male? or young female?).ti,ab,kf. | 108516 |
| 69 | (juvenile or young person? or young people or young adult$).ti,ab,kf. | 303236 |
| 70 | child$.ti,ab,kf. | 2139709 |
| 71 | or/55-70 [Set 5: Children and Adolescents] | 5675940 |
| 72 | 9 and (28 or 40 or 54) and 71 | 8533 |
| 73 | limit 72 to yr="2018-current" | 3031 |
| 74 | limit 73 to english language | 2986 |


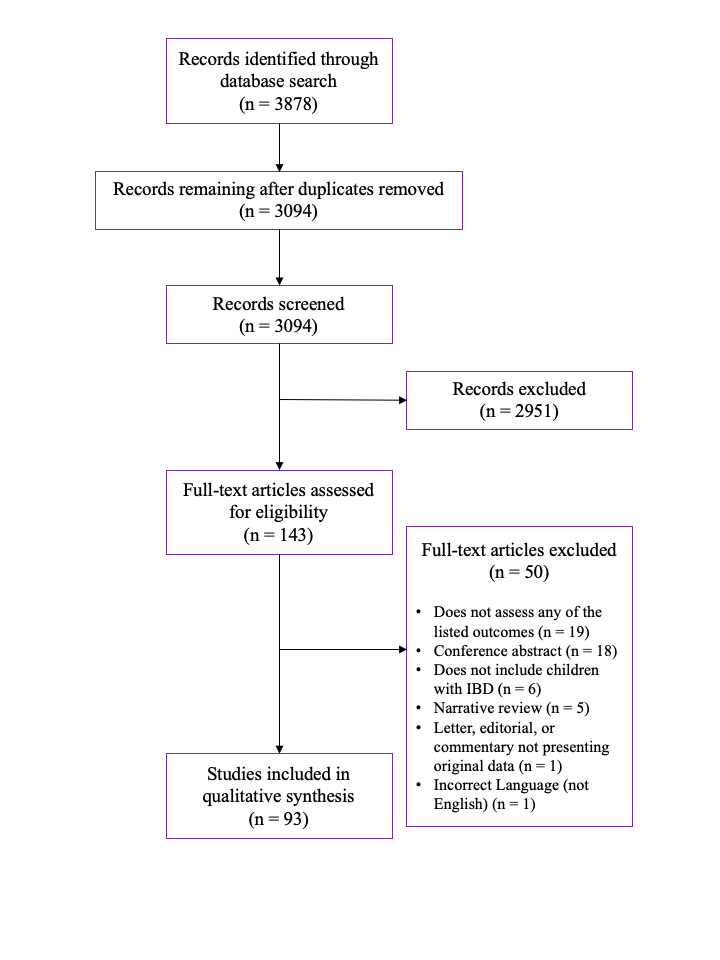


Figure 7: Flowchart detailing study selection for the Pediatric Growth, Development, and Education systematic review

Table 8.1: Transition from Pediatric to Adult Care inclusion and exclusion criteria

| **Inclusion Criteria** | **Exclusion Criteria** |
| --- | --- |
| - Describes some aspect of the transition from pediatric to adult care in youth with inflammatory bowel disease (IBD), Crohn’s disease (CD) or ulcerative colitis (UC). - This could include:   - Description of an intervention   - Evaluation of an intervention   - Patient perceptions of an intervention - This could also include   - Patient outcomes around the time of transition from adult to pediatric care (e.g., need for surgery or change in therapy)   - Health services utilization (e.g., clinic visits, hospitalizations, or emergency department visits)   *Note: Include studies where youth with IBD are included in studies involving youth with other chronic health conditions* | - Study does not include youth with IBD - Study does not describe or evaluate a transition program or evaluate patient outcomes at the transition period - Case series or report involving <5 IBD patients - Conference abstract - Letter, editorial, or commentary not presenting original data |

Table 8.2a: Transition from Pediatric to Adult Care search terms (MEDLINE), performed May 1, 2022

| **Ovid MEDLINE: Epub Ahead of Print, In-Process & Other Non-Indexed Citations, Ovid MEDLINE® Daily and Ovid MEDLINE® <1946-Present>** | | |
| --- | --- | --- |
| 1 | exp Inflammatory Bowel Diseases/ | 91149 |
| 2 | exp Colitis, Ulcerative/ | 38318 |
| 3 | exp Crohn Disease/ | 42437 |
| 4 | exp Proctitis/ | 3165 |
| 5 | exp Enteritis/ | 14141 |
| 6 | ("Inflammatory bowel disease$" or ulcerative colitis or Crohn$ or IBD or ileocolitis or ileitis or pancolitis or proctitis).af. | 131023 |
| 7 | or/1-6 [Set 1: IBD] | 145256 |
| 8 | "continuity of patient care"/ or patient handoff/ or patient transfer/ or transition to adult care/ or transitional care/ | 32878 |
| 9 | transition$.mp. | 501654 |
| 10 | "delivery of health care"/ or "delivery of health care, integrated"/ | 120891 |
| 11 | transfer$.mp. | 837244 |
| 12 | shared care.mp. | 1488 |
| 13 | or/8-12 [Set 2: Transition] | 1442594 |
| 14 | Adolescent/ | 2174285 |
| 15 | Young Adult/ | 990291 |
| 16 | Pediatrics/ | 57442 |
| 17 | Adolescent Medicine/ | 1559 |
| 18 | Adolescent Health/ | 1721 |
| 19 | Adolescent Health Services/ | 5837 |
| 20 | (teen$ or youth or adolescen$).af. | 2336987 |
| 21 | young adult?.af. | 1057937 |
| 22 | emerging adult?.af. | 2630 |
| 23 | p?ediatric$.af. | 1366945 |
| 24 | (young m#n or young wom#n or young male? or young female?).ti,ab,kf. | 78465 |
| 25 | (juvenile or young person? or young people or young adult$).ti,ab,kf. | 223282 |
| 26 | or/14-25 [Set 3: Age Group] | 3918392 |
| 27 | 7 and 13 and 26 | 797 |
| 28 | limit 27 to yr="2018-current" | 289 |
| 29 | limit 28 to english language | 283 |

Table 8.2b: Transition from Pediatric to Adult Care search terms (EMBASE), performed May 1, 2022

| **Embase Classic+Embase <1947 to 2022 April 29>** | | |
| --- | --- | --- |
| 1 | Inflammatory bowel disease/ | 44932 |
| 2 | exp Crohn disease/ | 105350 |
| 3 | exp ulcerative colitis/ | 89422 |
| 4 | proctitis/ | 7580 |
| 5 | ileitis/ | 7338 |
| 6 | pancolitis/ | 2208 |
| 7 | proctocolitis/ | 1351 |
| 8 | ("Inflammatory bowel disease$" or ulcerative colitis or Crohn$ or IBD or ileocolitis or ileitis or pancolitis or proctitis).af. | 236896 |
| 9 | or/1-8 [Set 1: IBD] | 237704 |
| 10 | transition to adult care/ | 2484 |
| 11 | transitional care/ | 4305 |
| 12 | integrated health care system/ | 12590 |
| 13 | patient care/ | 331278 |
| 14 | collaborative care team/ | 1567 |
| 15 | clinical handover/ | 2055 |
| 16 | transfer$.af. | 1084417 |
| 17 | shared care.af. | 2474 |
| 18 | transition$.af. | 645334 |
| 19 | or/10-18 [Set 2: Transition] | 2019642 |
| 20 | adolescent/ | 1808982 |
| 21 | young adult/ | 456679 |
| 22 | adolescence/ | 46961 |
| 23 | pediatrics/ | 94470 |
| 24 | adolescent health/ | 9799 |
| 25 | child health care/ | 39547 |
| 26 | (teen$ or youth or adolescen$).af. | 2070618 |
| 27 | young adult?.af. | 552140 |
| 28 | emerging adult?.af. | 3008 |
| 29 | p?ediatric.af. | 1127881 |
| 30 | (young m#n or young wom#n or young male? or young female?).ti,ab,kf. | 108405 |
| 31 | (juvenile or young person? or young people or young adult$).ti,ab,kf. | 302931 |
| 32 | or/20-31 [Set 3: Adolescence] | 3447826 |
| 33 | 9 and 19 and 32 | 1657 |
| 34 | limit 33 to yr="2018 -Current" | 709 |
| 35 | limit 34 to english language | 699 |


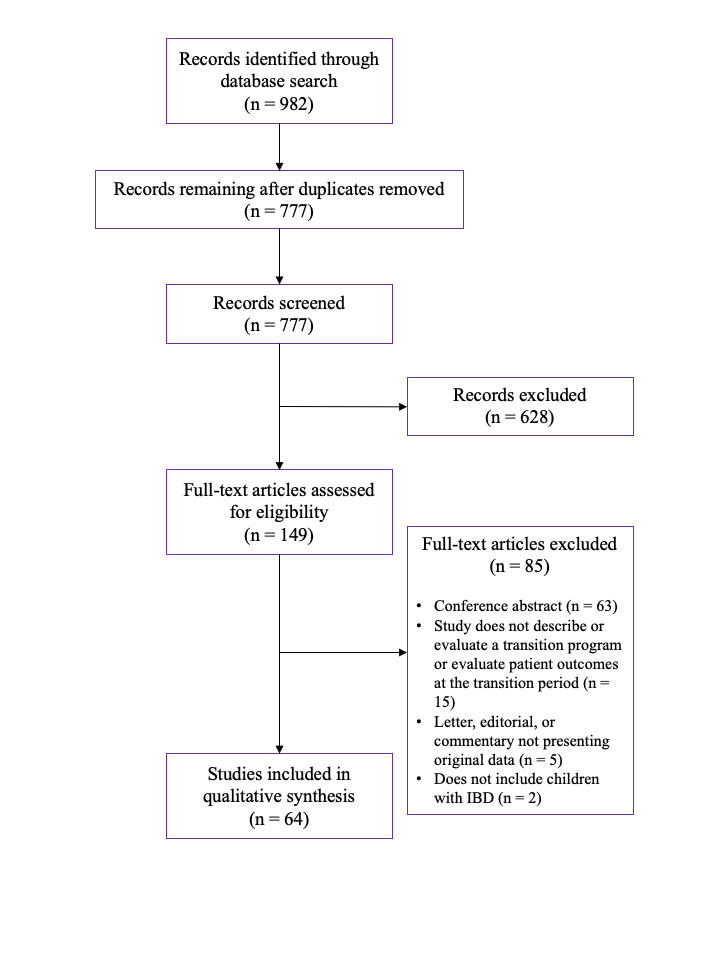


Figure 8: Flowchart detailing study selection for the Transition from Pediatric to Adult Care systematic review

Table 9.1: Age-related Comorbidities inclusion and exclusion criteria

| **Inclusion Criteria** | **Exclusion Criteria** |
| --- | --- |
| - Observational study, systematic review, narrative review, or clinical practice guideline; and - Includes seniors with inflammatory bowel disease, Crohn’s disease, and/or ulcerative colitis; and - Describes the incidence or prevalence of age-related chronic health condition, including diabetes, hypertension, cardiovascular disease, stroke, dementia, osteoporosis, arthritis, COPD, emphysema, kidney disease   *Note: Include studies where seniors with IBD are included in studies involving seniors with other chronic health conditions* | - Abstract/manuscript not in English - Conference abstract - Letter, editorial, or commentary not presenting original data - Case report or case series including <5 people with IBD - Does not include people with IBD - Does not report the rate of age-related chronic health condition - Qualitative study - Basic or translational science study |

Table 9.2a: Age-related Comorbidities search terms (MEDLINE), performed May 10, 2022

| **Ovid MEDLINE: Epub Ahead of Print, In-Process & Other Non-Indexed Citations, Ovid MEDLINE® Daily and Ovid MEDLINE® <1946-Present>** | | |
| --- | --- | --- |
| 1 | exp Inflammatory Bowel Diseases/ | 91402 |
| 2 | exp Colitis, Ulcerative/ | 38416 |
| 3 | exp Crohn Disease/ | 42513 |
| 4 | exp Proctitis/ | 3169 |
| 5 | exp Enteritis/ | 14168 |
| 6 | ("Inflammatory bowel disease$" or ulcerative colitis or Crohn$ or IBD or ileocolitis or ileitis or pancolitis or proctitis).mp. | 129181 |
| 7 | or/1-6 [Set 1: IBD] | 143585 |
| 8 | exp Dementia/ | 191288 |
| 9 | (dement$ or Alzheimer$).ti,ab,kf. | 255988 |
| 10 | cognitive impair$.ti,ab,kf. | 80777 |
| 11 | or/8-10 [Set 2: Dementia] | 339244 |
| 12 | exp Diabetes Mellitus/ | 479392 |
| 13 | diabet$.ti,ab,kf. | 723347 |
| 14 | exp Comorbidity/ | 123900 |
| 15 | ((age adj4 comorbid$) or mutli-morbid$ or multimorbid$).ti,ab,kf. | 26466 |
| 16 | exp Pulmonary Disease, Chronic Obstructive/ | 63359 |
| 17 | Multiple Chronic Conditions/ | 644 |
| 18 | ((respiratatory or pulmonary) adj3 dis$).ti,ab,kf. | 105856 |
| 19 | (emphysema or COPD).ti,ab,kf. | 76828 |
| 20 | exp heart diseases/ or exp cerebrovascular disorders/ or exp hypertension/ | 1823430 |
| 21 | cardiovascular diseases/ | 167231 |
| 22 | cardiovascular$ dis$.ti,ab,kf. | 216908 |
| 23 | ((myocardial or cerebr$) adj infarction$).ti,ab,kf. | 219297 |
| 24 | strok$.ti,ab,kf. | 293185 |
| 25 | hyperten$.ti,ab,kf. | 484543 |
| 26 | heart fail$.ti,ab,kf. | 200638 |
| 27 | atrial fibrillation.ti,ab,kf. | 84000 |
| 28 | exp Osteoporosis/ | 60413 |
| 29 | osteopor$.ti,ab,kf. | 87391 |
| 30 | osteopeni$.ti,ab,kf. | 12058 |
| 31 | exp Kidney Diseases/ | 554163 |
| 32 | ((kidney or renal) adj3 (fail$ or insufficien$)).ti,ab,kf. | 130577 |
| 33 | exp Joint Diseases/ | 412838 |
| 34 | (osteoarthritis or arthritis or arthropath$).ti,ab,kf. | 269866 |
| 35 | or/12-24 [Set 3: Other age-related comorbidities] | 3012105 |
| 36 | limit 35 to "reviews (best balance of sensitivity and specificity)" | 457274 |
| 37 | 7 and (11 or 36) | 2537 |
| 38 | 37 not ((exp animal/ or exp invertebrate/ or animal experiment/ or animal model/ or exp plant/ or exp fungus/) not exp human/) | 2485 |
| 39 | limit 38 to yr="2018-current" | 874 |
| 40 | limit 39 to english language | 859 |

Table 9.2b: Age-related Comorbidities search terms (EMBASE), performed May 10, 2022

| **Embase Classic+Embase <1947 to 2022 May 06>** | | |
| --- | --- | --- |
| 1 | Inflammatory bowel disease/ | 45077 |
| 2 | exp Crohn disease/ | 105411 |
| 3 | exp ulcerative colitis/ | 89491 |
| 4 | proctitis/ | 7581 |
| 5 | ileitis/ | 7344 |
| 6 | pancolitis/ | 2209 |
| 7 | proctocolitis/ | 1353 |
| 8 | ("Inflammatory bowel disease*" or ulcerative colitis or Crohn* or IBD or ileocolitis or ileitis or pancolitis or proctitis).af. | 237168 |
| 9 | or/1-8 [Set 1: IBD] | 237978 |
| 10 | exp dementia/ | 411928 |
| 11 | (dement$ or Alzheimer$).ti,ab,kf. | 360084 |
| 12 | cognitive impair$.ti,ab,kf. | 124332 |
| 13 | or/10-12 [Set 2: Dementia] | 547621 |
| 14 | exp diabetes mellitus/ | 1148187 |
| 15 | diabet$.ti,ab,kf. | 1122522 |
| 16 | comorbidity/ | 334682 |
| 17 | multiple chronic conditions/ | 5890 |
| 18 | ((age adj4 comorbid$) or mutli-morbid$ or multimorbid$).ti,ab,kf. | 45300 |
| 19 | obstructive lung disease/ or chronic obstructive lung disease/ | 157276 |
| 20 | exp lung emphysema/ | 28502 |
| 21 | ((respiratatory or pulmonary) adj3 dis$).ti,ab,kf. | 163203 |
| 22 | (emphysema or COPD).ti,ab,kf. | 135995 |
| 23 | exp heart disease/ or cardiovascular disease/ | 2429057 |
| 24 | exp cerebrovascular disease/ | 763832 |
| 25 | exp hypertension/ | 883376 |
| 26 | cardiovascular$ dis$.ti,ab,kf. | 316138 |
| 27 | ((myocardial or cerebr$) adj infarction$).ti,ab,kf. | 343017 |
| 28 | strok$.ti,ab,kf. | 474702 |
| 29 | hyperten$.ti,ab,kf. | 783950 |
| 30 | heart fail$.ti,ab,kf. | 340153 |
| 31 | atrial fibrillation.ti,ab,kf. | 151897 |
| 32 | exp osteoporosis/ | 148903 |
| 33 | osteopor$.ti,ab,kf. | 141318 |
| 34 | osteopeni$.ti,ab,kf. | 20698 |
| 35 | exp kidney disease/ | 1141204 |
| 36 | ((kidney or renal) adj3 (fail$ or insufficien$)).ti,ab,kf. | 204037 |
| 37 | exp arthropathy/ | 916034 |
| 38 | (osteoarthritis or arthritis or arthropath$).ti,ab,kf. | 412390 |
| 39 | or/14-38 [Set 3: Other age-related comorbidities] | 6862413 |
| 40 | limit 39 to "reviews (best balance of sensitivity and specificity)" | 838186 |
| 41 | 9 and (13 or 40) | 13230 |
| 42 | 41 not ((exp animal/ or exp invertebrate/ or nonhuman/ or animal experiment/ or animal tissue/ or animal model/ or exp plant/ or exp fungus/) not (exp human/ or human tissue/)) | 12895 |
| 43 | limit 42 to yr="2018-current" | 4177 |
| 44 | limit 43 to english language | 4123 |
| 45 | limit 44 to conference abstract | 325 |
| 46 | 44 not 45 | 3798 |


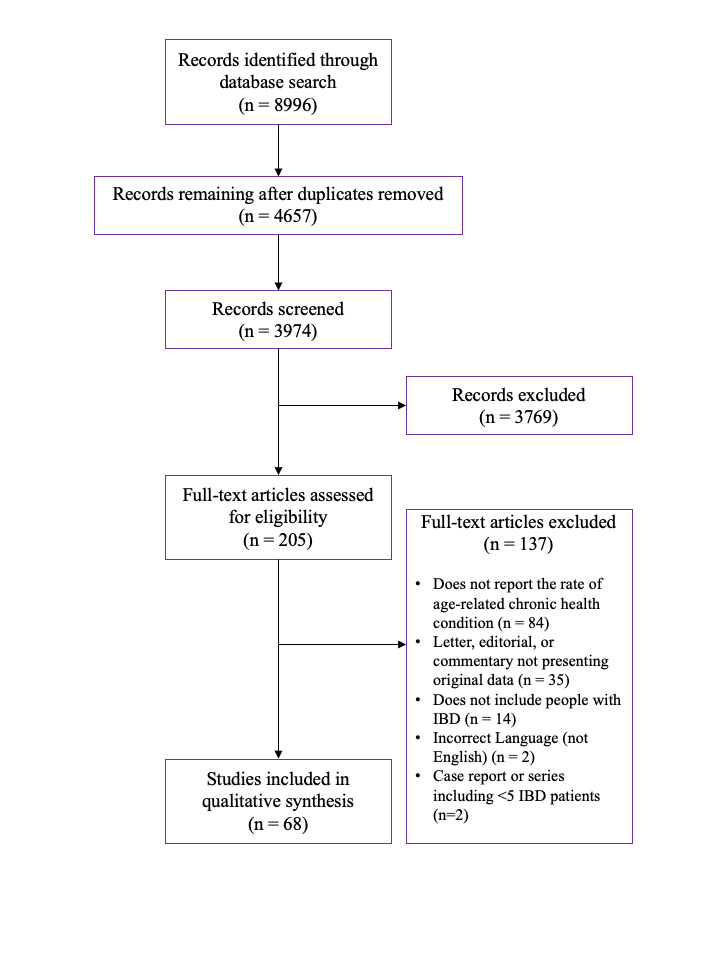


Figure 9: Flowchart detailing study selection for the Age-related Comorbidities systematic review

Table 10.1: Treatment of Seniors inclusion and exclusion criteria

| **Inclusion Criteria** | **Exclusion Criteria** |
| --- | --- |
| Among people with inflammatory bowel disease (IBD), Crohn’s disease (CD), or ulcerative colitis (UC) who are 65 years of age or older, report at least one of the following:   - Observational study or randomized controlled trial describing the effectiveness or efficacy of a medication used to treat IBD; or - Observational study or randomized controlled trial describing the safety of a medication used to treat IBD, including but not limited to adverse events and infection; or - Systematic or narrative review summarizing the efficacy, effectiveness, safety, or infection risk in seniors with IBD; or - Clinical practice guideline discussing treatment approaches in elderly IBD.   *Note: Include studies if data on seniors are available as a subgroup of a larger study.*  *Note: Include studies where seniors with IBD are included in studies involving seniors with other chronic health conditions.*  *Note: Medications used to treat IBD, CD, and UC include biologic therapy (anti-TNFs: infliximab, adalimumab, golimumab, certolizumab; vedolizumab; ustekinumab), corticosteroids (systemic steroids or budesonide), immunomodulators (azathioprine, 6-mercaptopurine, or methotrexate), mesalamine (5-ASA, sulfasalazine, etc), JAK inhibitors (tofacitinib).* | - Conference abstract - Manuscript is not in English - Case series or report including <5 IBD patients - Letter, editorial, or commentary not presenting original data - Qualitative study - Does not include people with IBD - Does not include data specific to seniors - Does not report on efficacy/effectiveness, safety, or infection risk |

Table 10.2a: Treatment of Seniors search terms (MEDLINE), performed May 10, 2022

| **Ovid MEDLINE: Epub Ahead of Print, In-Process & Other Non-Indexed Citations, Ovid MEDLINE® Daily and Ovid MEDLINE® <1946-Present>** | | |
| --- | --- | --- |
| 1 | exp Inflammatory Bowel Diseases/ | 91402 |
| 2 | exp Colitis, Ulcerative/ | 38416 |
| 3 | exp Crohn Disease/ | 42513 |
| 4 | exp Proctitis/ | 3169 |
| 5 | exp Enteritis/ | 14168 |
| 6 | ("Inflammatory bowel disease*" or ulcerative colitis or Crohn* or IBD or ileocolitis or ileitis or pancolitis or proctitis).mp. | 129181 |
| 7 | or/1-6 [Set 1: IBD] | 143585 |
| 8 | Tumor Necrosis Factor-alpha/ or Tumor Necrosis Factor Inhibitors/ | 134329 |
| 9 | Infliximab/ | 11525 |
| 10 | Adalimumab/ | 6464 |
| 11 | Certolizumab Pegol/ | 705 |
| 12 | (infliximab or adalimumab or certolizumab or golimumab).ti,ab,kf. | 19248 |
| 13 | vedolizumab.ti,ab,kf. | 1337 |
| 14 | Ustekinumab/ | 1507 |
| 15 | ustekinumab.ti,ab,kf. | 2469 |
| 16 | Mesalamine/ or Sulfasalazine/ | 7499 |
| 17 | (aminosalicylic acid or aminosalicylate).ti,ab,kf. | 6509 |
| 18 | (mesalazine or mesalamine).ti,ab,kf. | 2785 |
| 19 | (olsalazine or balsalazide or sulfasalazine).ti,ab,kf. | 3878 |
| 20 | exp Adrenal Cortex Hormones/ | 415623 |
| 21 | (prednisone or prednisolone or corticosteroid* or steroid*).ti,ab,kf. | 397533 |
| 22 | Azathioprine/ or Mercaptopurine/ | 20234 |
| 23 | (mercaptopurine or 6*MP or 6MP or azathioprine or thiopurine).ti,ab,kf. | 21195 |
| 24 | Methotrexate/ | 40193 |
| 25 | methotrexate.ti,ab,kf. | 45287 |
| 26 | immunomodulator$.ti,ab,kf. | 55511 |
| 27 | Janus Kinase Inhibitors/ | 1055 |
| 28 | tofacitinib.ti,ab,kf. | 2095 |
| 29 | Cyclosporine/ | 30206 |
| 30 | cyclosporine.ti,ab,kf. | 31883 |
| 31 | or/8-30 [Set 2: Therapeutics] | 959275 |
| 32 | exp Aged/ | 3399293 |
| 33 | (senior$ or elder$).ti,ab,kf. | 334510 |
| 34 | older adult$.ti,ab,kf. | 103903 |
| 35 | or/32-34 [Set 3: Seniors] | 3532280 |
| 36 | 7 and 31 and 35 | 4156 |
| 37 | limit 36 to yr="2018-current" | 756 |
| 38 | limit 37 to english language | 742 |

Table 10.2b: Treatment of Seniors search terms (EMBASE), performed May 10, 2022

| **Embase Classic+Embase <1947 to 2022 May 06>** | | |
| --- | --- | --- |
| 1 | Inflammatory bowel disease/ | 45077 |
| 2 | exp Crohn disease/ | 105411 |
| 3 | exp ulcerative colitis/ | 89491 |
| 4 | proctitis/ | 7581 |
| 5 | ileitis/ | 7344 |
| 6 | pancolitis/ | 2209 |
| 7 | proctocolitis/ | 1353 |
| 8 | ("Inflammatory bowel disease*" or ulcerative colitis or Crohn* or IBD or ileocolitis or ileitis or pancolitis or proctitis).af. | 237168 |
| 9 | or/1-8 [Set 1: IBD] | 237978 |
| 10 | tumor necrosis factor/ | 188713 |
| 11 | tumor necrosis factor antibody/ | 5745 |
| 12 | infliximab/ | 57440 |
| 13 | adalimumab/ | 40129 |
| 14 | certolizumab pegol/ | 8114 |
| 15 | golimumab/ | 8702 |
| 16 | (infliximab or adalimumab or certolizumab or golimumab).ti,ab,kf. | 43372 |
| 17 | vedolizumab/ | 5844 |
| 18 | vedolizumab.ti,ab,kf. | 3980 |
| 19 | ustekinumab/ | 9961 |
| 20 | ustekinumab.ti,ab,kf. | 5672 |
| 21 | mesalazine/ | 20404 |
| 22 | salazosulfapyridine/ | 28032 |
| 23 | (aminosalicylic acid or aminosalicylate).ti,ab,kf. | 6108 |
| 24 | (mesalazine or mesalamine).ti,ab,kf. | 5590 |
| 25 | (olsalazine or balsalazide or sulfasalazine).ti,ab,kf. | 6528 |
| 26 | olsalazine/ | 1501 |
| 27 | balsalazide/ | 961 |
| 28 | glucocorticoid/ or corticosteroid/ or prednisone/ | 542750 |
| 29 | (prednisone or prednisolone or corticosteroid* or steroid*).ti,ab,kf. | 619725 |
| 30 | azathioprine/ or mercaptopurine/ | 124998 |
| 31 | (mercaptopurine or 6*MP or 6MP or azathioprine or thiopurine).ti,ab,kf. | 38230 |
| 32 | methotrexate/ | 200298 |
| 33 | methotrexate.ti,ab,kf. | 78054 |
| 34 | immunomodulator$.ti,ab,kf. | 83942 |
| 35 | janus kinase inhibitor/ or tofacitinib/ | 10611 |
| 36 | tofacitinib.ti,ab,kf. | 4436 |
| 37 | cyclosporine/ | 22472 |
| 38 | cyclosporine.ti,ab,kf. | 48128 |
| 39 | or/10-38 [Set 2: Therapeutics] | 1454920 |
| 40 | exp aged/ | 3541430 |
| 41 | (senior$ or elder$).ti,ab,kf. | 488352 |
| 42 | older adult$.ti,ab,kf. | 130551 |
| 43 | or/40-42 [Set 3: Seniors] | 3717455 |
| 44 | 9 and 39 and 43 | 6897 |
| 45 | limit 44 to yr="2018-current" | 2281 |
| 46 | limit 45 to english language | 2262 |
| 47 | limit 46 to conference abstract | 611 |
| 48 | 46 not 47 | 1651 |


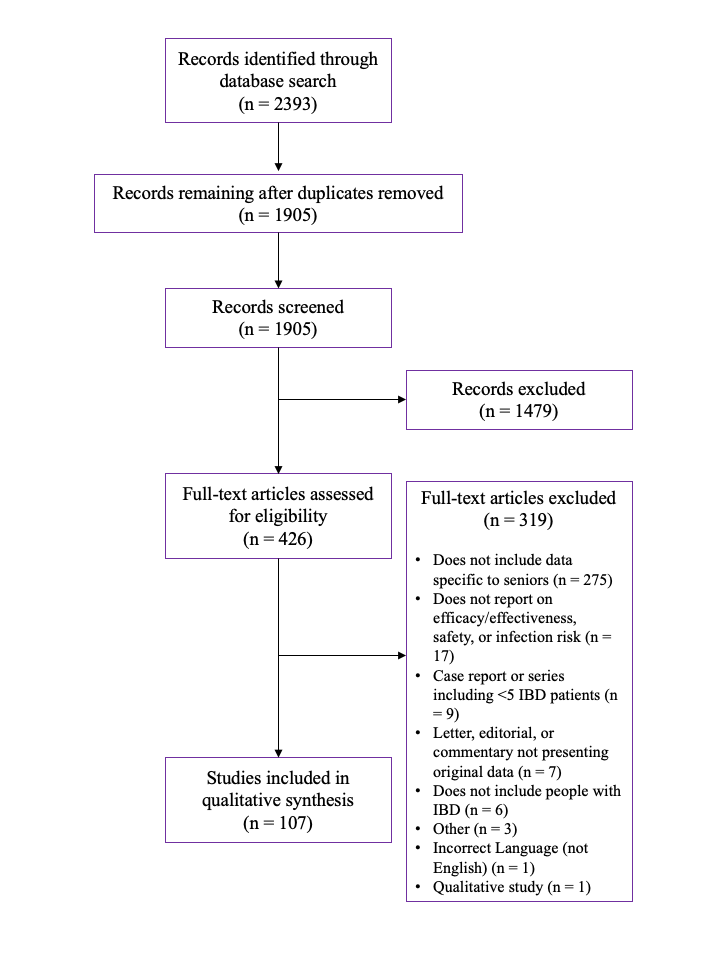


Figure 10: Flowchart detailing study selection for the Treatment of Seniors systematic review

Table 11.1: Vaccines for Seniors inclusion and exclusion criteria

| **Inclusion Criteria** | **Exclusion Criteria** |
| --- | --- |
| Among people with inflammatory bowel disease (IBD), Crohn’s disease (CD), or ulcerative colitis (UC) who are 65 years of age or older, report at least one of the following:   - Vaccine effectiveness or efficacy (observational study, randomized controlled trial, systematic review, or narrative review) - Adverse events following vaccination (observational study, randomized controlled trial, systematic review, or narrative review) - Rates of vaccine uptake (observational study, systematic review, or narrative review)   The following are also eligible for inclusion:   - Clinical practice guidelines providing recommendations for vaccinating seniors with IBD - Qualitative studies describing barriers to vaccination or vaccine hesitancy in seniors with IBD (or review article summarizing these studies)   *Note: Include studies if data on seniors are available as a subgroup of a larger study*  *Note: Include studies where seniors with IBD are included in studies involving seniors with other chronic health conditions*  *Note: Observational studies include cohort, case-control, or cross-sectional studies* | - Does not include seniors with IBD - Does not describe any vaccine-related outcomes - Does not include data specific to seniors - Case series or report involving <5 IBD patients - Letter, commentary, or editorial that does not include original data - Conference abstract - Study is not written in English - Other |

Table 11.2a: Vaccines for Seniors search terms (MEDLINE), performed May 14, 2022

| **Ovid MEDLINE: Epub Ahead of Print, In-Process & Other Non-Indexed Citations, Ovid MEDLINE® Daily and Ovid MEDLINE® <1946-Present>** | | |
| --- | --- | --- |
| 1 | exp Inflammatory Bowel Diseases/ | 91393 |
| 2 | exp Colitis, Ulcerative/ | 38408 |
| 3 | exp Crohn Disease/ | 42519 |
| 4 | exp Proctitis/ | 3170 |
| 5 | exp Enteritis/ | 14168 |
| 6 | ("Inflammatory bowel disease*" or ulcerative colitis or Crohn* or IBD or ileocolitis or ileitis or pancolitis or proctitis).mp. | 129160 |
| 7 | or/1-6 [Set 1: IBD] | 143566 |
| 8 | exp Aged/ | 3398776 |
| 9 | (senior$ or elder$).ti,ab,kf. | 334514 |
| 10 | older adult$.ti,ab,kf. | 103988 |
| 11 | or/8-10 [Set 2: Seniors] | 3531752 |
| 12 | exp Vaccines/ | 259217 |
| 13 | exp Immunization/ | 197365 |
| 14 | (vaccine$ or vaccinat$ or immuni$ or inoculat$).ti,ab,kf. | 733486 |
| 15 | or/12-14 [Set 3: Vaccines] | 826401 |
| 16 | 7 and 11 and 15 | 340 |
| 17 | limit 16 to yr="2018-current" | 94 |
| 18 | limit 17 to english language | 93 |

Table 11.2b: Vaccines for Seniors search terms (EMBASE), performed May 14, 2022

| **Embase Classic+Embase <1947 to 2022 May 13>** | | |
| --- | --- | --- |
| 1 | Inflammatory bowel disease/ | 45199 |
| 2 | exp Crohn disease/ | 105476 |
| 3 | exp ulcerative colitis/ | 89566 |
| 4 | proctitis/ | 7586 |
| 5 | ileitis/ | 7350 |
| 6 | pancolitis/ | 2212 |
| 7 | proctocolitis/ | 1353 |
| 8 | ("Inflammatory bowel disease*" or ulcerative colitis or Crohn* or IBD or ileocolitis or ileitis or pancolitis or proctitis).af. | 237400 |
| 9 | or/1-8 [Set 1: IBD] | 238210 |
| 10 | exp aged/ | 3545632 |
| 11 | (senior$ or elder$).ti,ab,kf. | 488939 |
| 12 | older adult$.ti,ab,kf. | 131324 |
| 13 | or/10-12 [Set 2: Seniors] | 3721826 |
| 14 | exp vaccine/ | 415691 |
| 15 | exp immunization/ | 366165 |
| 16 | (vaccine$ or vaccinat$ or immuni$ or inoculat$).ti,ab,kf. | 926418 |
| 17 | or/14-16 [Set 3: Vaccination] | 1079486 |
| 18 | 9 and 13 and 17 | 493 |
| 19 | limit 18 to yr="2018-current" | 202 |
| 20 | limit 19 to english language | 200 |
| 21 | limit 20 to conference abstract | 46 |
| 22 | 20 not 21 | 154 |


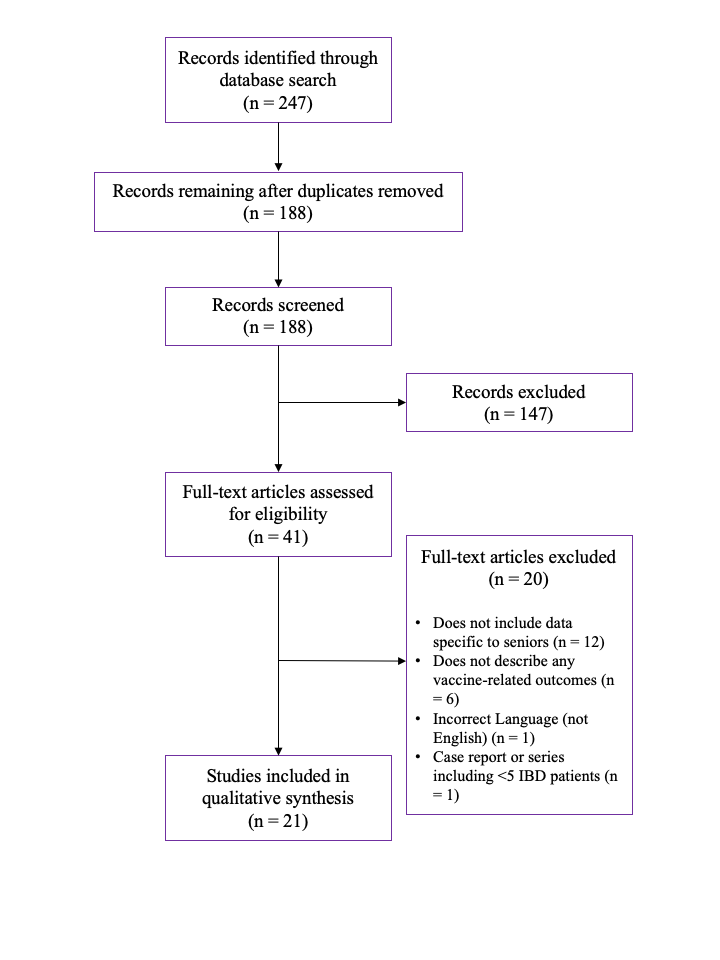


Figure 11: Flowchart detailing study selection for the Vaccines for Seniors systematic review

Table 12.1: Biosimilars inclusion and exclusion criteria

| **Inclusion Criteria** | **Exclusion Criteria** |
| --- | --- |
| - Observational study or randomized controlled trial assessing outcomes of patients with inflammatory bowel disease (IBD), Crohn’s disease (CD), or ulcerative colitis (UC) on a biosimilar medication or compares outcomes in patients receiving a biosimilar and original biologic. Studies can include those in which a patient starts on one medication then switches to another or where patients are on the same medication throughout the course of the study; or - Systematic or narrative review summarizing outcomes of IBD/CD/UC patients receiving biosimilars or comparing outcomes of IBD/CD/UC patients receiving biosimilars and originator biologics; or - Clinical practice guidelines outlining the use of biosimilars in patients with IBD, CD, or UC; - Study describing the economic impact (e.g., cost-benefit or cost-effectiveness study) of using biosimilars to manage patients with IBD, CD, or UC; or - Qualitative study describing patients’ perspectives of any aspect of biosimilar utilization (patient preferences, forced switch policies, etc.)   *Note: Include studies where people with IBD are included in studies involving people with other chronic health conditions.* | - Conference abstract - Manuscript not written in English - Case series or report including <5 IBD patients - Does not include patients with IBD - Basic or translational science study - Assesses outcomes in patients receiving originator biologics, but not biosimilars - Compares intravenous and subcutaneous administration of an originator biologic without assessing biosimilars - Other reason for exclusion |

Table 12.2a: Biosimilars search terms (MEDLINE), performed May 12, 2022

| **Ovid MEDLINE: Epub Ahead of Print, In-Process & Other Non-Indexed Citations, Ovid MEDLINE® Daily and Ovid MEDLINE® <1946-Present>** | | |
| --- | --- | --- |
| 1 | exp Inflammatory Bowel Diseases/ | 91345 |
| 2 | exp Colitis, Ulcerative/ | 38392 |
| 3 | exp Crohn Disease/ | 42502 |
| 4 | exp Proctitis/ | 3169 |
| 5 | exp Enteritis/ | 14167 |
| 6 | ("Inflammatory bowel disease$" or ulcerative colitis or Crohn$ or IBD or ileocolitis or ileitis or pancolitis or proctitis).mp. | 129112 |
| 7 | or/1-6 [Set 1: IBD] | 143518 |
| 8 | Biosimilar Pharmaceuticals/ | 2943 |
| 9 | biosimilar$.ti,ab,kf. | 4564 |
| 10 | origina$ biologic$.ti,ab,kf. | 357 |
| 11 | or/8-10 [Set 2: Biosimilar] | 4981 |
| 12 | 7 and 11 | 540 |
| 13 | limit 12 to yr="2018-current" | 344 |
| 14 | limit 13 to english language | 339 |

Table 12.2b: Biosimilars search terms (EMBASE), performed May 12, 2022

| **Embase Classic+Embase <1947 to 2022 May 11>** | | |
| --- | --- | --- |
| 1 | Inflammatory bowel disease/ | 45151 |
| 2 | exp Crohn disease/ | 105456 |
| 3 | exp ulcerative colitis/ | 89537 |
| 4 | proctitis/ | 7582 |
| 5 | ileitis/ | 7344 |
| 6 | pancolitis/ | 2211 |
| 7 | proctocolitis/ | 1353 |
| 8 | ("Inflammatory bowel disease*" or ulcerative colitis or Crohn* or IBD or ileocolitis or ileitis or pancolitis or proctitis).af. | 237312 |
| 9 | or/1-8 [Set 1: IBD] | 238122 |
| 10 | biosimilar agent/ | 5912 |
| 11 | biosimilar$.ti,ab,kf. | 9227 |
| 12 | origina$ biologic$.ti,ab,kf. | 573 |
| 13 | or/10-12 [Set 2: Biosimilars] | 10618 |
| 14 | 9 and 13 | 1460 |
| 15 | limit 14 to yr="2018-current" | 867 |
| 16 | limit 15 to english language | 848 |
| 17 | limit 16 to conference abstract | 362 |
| 18 | 16 not 17 | 486 |


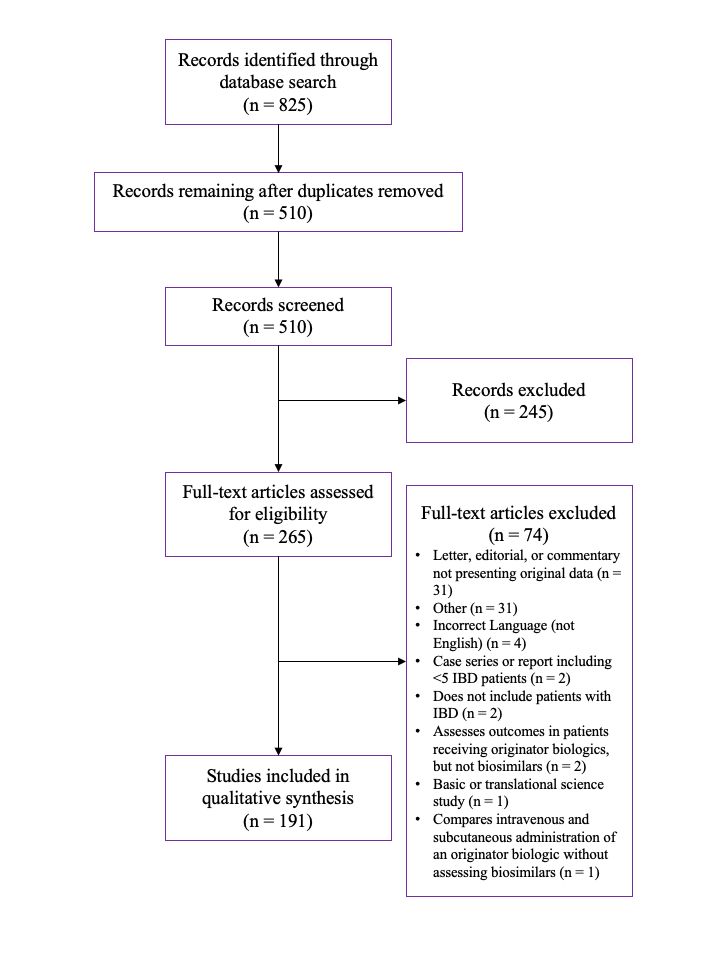


Figure 12: Flowchart detailing study selection for the Biosimilars systematic review

Table 13.1: Management Strategies inclusion and exclusion criteria

| **Inclusion Criteria** | **Exclusion Criteria** |
| --- | --- |
| - Observational study studying the impact of therapeutic drug monitoring in patients with inflammatory bowel disease (IBD), Crohn’s disease (CD), or ulcerative colitis (UC) on biologic therapy; or - Randomized controlled trial evaluating the impact of therapeutic drug monitoring compared to standard of care in patients with IBD; or - Economic evaluation (e.g., cost-benefit or cost-effectiveness study) describing the value of utilizing therapeutic drug monitoring in clinical care; or - Clinical practice guidelines providing recommendations for integrating therapeutic drug monitoring into routine clinical practice; or - Observational study describing the association between trough levels or anti-drug antibodies and outcomes in patients with IBD, CD, or UC; or - Secondary analysis of a randomized controlled trial describing the association between trough levels or anti-drug antibodies and outcomes in patients with IBD, CD, or UC; or - Observational study describing differences in “step up” versus “top down” therapeutic approaches in patients with IBD, CD, or UC; or - Randomized controlled trial comparing “step up” and “top down” therapeutic approaches in patients with IBD, CD, or UC; or - Economic evaluation (e.g., cost-benefit or cost-effectiveness study) describing the value of “step up” versus “top down” therapy in people with IBD, CD, or UC; or - Observational study describing the association between “treat-to-target” disease management approaches and outcomes in people with IBD, CD, or UC; - Randomized controlled trial comparing “treat-to-target” disease management approaches with standard of care in people with IBD, CD, or UC; - Economic evaluation (e.g., cost-benefit or cost-effectiveness study) describing the value of “treat-to-target” approaches in people with IBD, CD, or UC; or - Observational study describing the association between dose escalation or de-escalation and outcomes in patients with IBD, CD, or UC; or - Randomized controlled trial assessing the impact of dose escalation or de-escalation and outcomes in patients with IBD, CD, or UC; or - Economic evaluation (e.g., cost-benefit or cost-effectiveness study) describing the impact of dose escalation or dose de-escalation in people with IBD, CD, or UC; or - Observational study comparing outcomes in patients with IBD, CD, or UC early versus late in their disease course or describing the association between time to therapy and outcomes in patients with IBD, CD, or UC; or - Secondary analysis of randomized controlled trial describing the association between receiving therapy later in the disease course and outcomes in patients with IBD, CD, or UC; or - Observational study describing the impact of insurance-related delays on access to therapy or outcomes in people with IBD, CD, or UC; or - Observational study describing the association between fecal calprotectin levels and outcomes in people with IBD, CD, or UC; or - Observational study describing the association between the use of fecal calprotectin levels and outcomes in people with IBD, CD, or UC; or - Secondary analysis of randomized controlled trial describing the association between fecal calprotectin levels and outcomes in people with IBD, CD, or UC; or - Narrative or systematic review synthesizing evidence on any of the above topics; or - Clinical practice guidelines outlining how any of the above disease management strategies or tools used to drive these management strategies should be incorporated into clinical care.   *Note: Outcomes of relevant studies could include (but are not limited to) disease activity indices, endoscopic disease activity or mucosal healing, need for hospitalization or surgery, need for systemic steroids, or discontinuation of therapy.*  *Note: Treat-to-target strategies include any that modifies IBD therapy based on test results, including endoscopy, imaging, fecal calprotectin, disease indices, or other measure of monitoring disease activity in people with IBD.* | - Conference abstract - Manuscript not written in English - Case report or case series with <5 IBD patients - Editorial, commentary, or letter to the editor that does not include any original data - Basic or translational science study - Does not include people with IBD - Does not assess IBD outcomes associated with any of the above management strategies or tools to aid in management strategies |

Table 13.2a: Management Strategies search terms (MEDLINE), performed May 24, 2022

| **Ovid MEDLINE: Epub Ahead of Print, In-Process & Other Non-Indexed Citations, Ovid MEDLINE® Daily and Ovid MEDLINE® <1946-Present>** | | |
| --- | --- | --- |
| 1 | exp Inflammatory Bowel Diseases/ | 91523 |
| 2 | exp Colitis, Ulcerative/ | 38452 |
| 3 | exp Crohn Disease/ | 42572 |
| 4 | exp Proctitis/ | 3173 |
| 5 | exp Enteritis/ | 14181 |
| 6 | ("Inflammatory bowel disease$" or ulcerative colitis or Crohn$ or IBD or ileocolitis or ileitis or pancolitis or proctitis).mp. | 129361 |
| 7 | or/1-6 [Set 1: IBD] | 143777 |
| 8 | Drug Monitoring/ | 23148 |
| 9 | ("therapeutic drug" adj5 (monitor$ or level$)).ti,ab,kf. | 9968 |
| 10 | ((drug or trough) adj (concentration$ or level$)).ti,ab,kf. | 37331 |
| 11 | anti-drug antibod$.ti,ab,kf. | 1255 |
| 12 | (dose adj3 (escalat$ or de-escalat$)).ti,ab,kf. | 17320 |
| 13 | (dose adj3 titrat$).ti,ab,kf. | 4254 |
| 14 | ("step up" or "top down").ti,ab,kf. | 21190 |
| 15 | (earl$ adj3 (therap$ or biologic$)).ti,ab,kf. | 29365 |
| 16 | treat to target.ti,ab,kf. | 1646 |
| 17 | insurance, pharmaceutical services/ or prior authorization/ | 4234 |
| 18 | ((delay$ or prior) adj5 (insuran$ or authoriz$ or authoris$)).ti,ab,kf. | 1416 |
| 19 | f?ecal calprotectin.ti,ab,kf. | 2295 |
| 20 | or/8-19 [Set 2: Management] | 142747 |
| 21 | 7 and 20 | 4129 |
| 22 | limit 21 to yr="2018-current" | 2003 |
| 23 | limit 22 to english language | 1976 |
| 24 | 23 not ((exp animal/ or exp invertebrate/ or animal experiment/ or animal model/ or exp plant/ or exp fungus/) not exp human/) | 1958 |

Table 13.2b: Management Strategies search terms (EMBASE), performed May 24, 2022

| **Embase Classic+Embase <1947 to 2022 May 23>** | | |
| --- | --- | --- |
| 1 | Inflammatory bowel disease/ | 45471 |
| 2 | exp Crohn disease/ | 105601 |
| 3 | exp ulcerative colitis/ | 89699 |
| 4 | proctitis/ | 7592 |
| 5 | ileitis/ | 7364 |
| 6 | pancolitis/ | 2220 |
| 7 | proctocolitis/ | 1354 |
| 8 | ("Inflammatory bowel disease*" or ulcerative colitis or Crohn* or IBD or ileocolitis or ileitis or pancolitis or proctitis).af. | 237899 |
| 9 | or/1-8 [Set 1: IBD] | 238709 |
| 10 | drug monitoring/ | 56895 |
| 11 | ("therapeutic drug" adj5 (monitor$ or level$)).ti,ab,kf. | 15936 |
| 12 | ((drug or trough) adj (concentration$ or level$)).ti,ab,kf. | 55819 |
| 13 | anti-drug antibod$.ti,ab,kf. | 3107 |
| 14 | (dose adj3 (escalat$ or de-escalat$)).ti,ab,kf. | 38783 |
| 15 | (dose adj3 titrat$).ti,ab,kf. | 7712 |
| 16 | ("step up" or "top down").ti,ab,kf. | 24995 |
| 17 | (earl$ adj3 (therap$ or biologic$)).ti,ab,kf. | 47603 |
| 18 | treat to target.ti,ab,kf. | 3895 |
| 19 | health insurance/ or prior authorization/ | 140866 |
| 20 | ((delay$ or prior) adj5 (insuran$ or authoriz$ or authoris$)).ti,ab,kf. | 2966 |
| 21 | f?ecal calprotectin.ti,ab,kf. | 6203 |
| 22 | or/10-21 [Set 2: Management] | 380009 |
| 23 | 9 and 22 | 12032 |
| 24 | limit 23 to yr="2018-current" | 5648 |
| 25 | limit 24 to english language | 5561 |
| 26 | limit 25 to conference abstract | 3177 |
| 27 | 25 not 26 | 2384 |
| 28 | 27 not ((exp animal/ or exp invertebrate/ or nonhuman/ or animal experiment/ or animal tissue/ or animal model/ or exp plant/ or exp fungus/) not (exp human/ or human tissue/)) | 2354 |


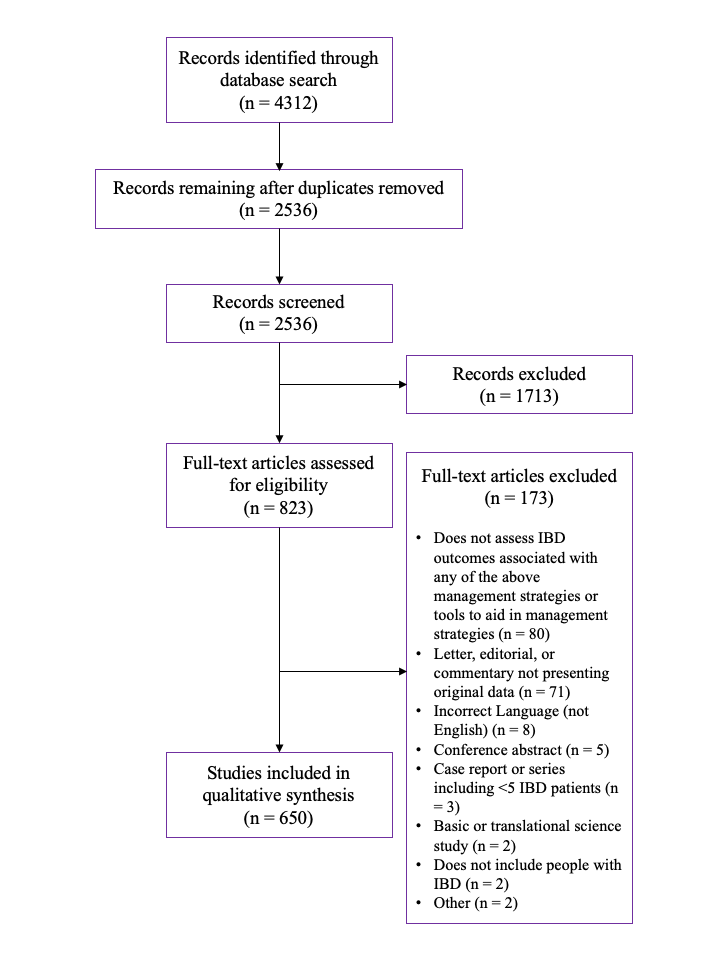


Figure 13: Flowchart detailing study selection for the Management Strategies systematic review
