# Supplementary material for: The 2023 Impact of Inflammatory Bowel Disease in Canada: Executive Summary
Source: J Can Assoc Gastroenterol. 2023 Jun 1;6(Suppl 2):S1–8. doi: 10.1093/jcag/gwad003 (PMC10478799; doi:10.1093/jcag/gwad003)
Supplement: gwad003_suppl_Supplementary_Material_2 [file gwad003_suppl_supplementary_material_2.pdf]

Impact of IBD in Canada Report - Patient and Family Partner Feedback

Introduction

Thank you for taking your time to provide valuable and insightful feedback on a chapter from our forthcoming Impact of Inflammatory Bowel Disease in Canada 2023 report.

You have been provided a draft chapter that will appear in a scientific report to be published in the Journal of the Canadian Association of Gastroenterology. Based on the feedback you provide, we will edit the chapter to be specifically relevant to those in the IBD community, with language geared towards a non-specialist audience.

Please review the consent form on the next page and answer the questions to the best of your ability. We will invite you to provide additional feedback orally at a consensus meeting in 2023, after we incorporate your initial feedback from this survey.

Impact of IBD in Canada Report - Patient and Family Partner Feedback

Online Consent

Dear Patient/Family partner,

Thank you very much for volunteering to contribute to the 2023 Impact of Inflammatory Bowel Disease(IBD) in Canada Report! Your experiences as a person living with IBD or as a family member or caregiver of an individual with IBD will enhance our interpretation of the most current evidence.

Introduction

The Impact of IBD in Canada report is a wide-ranging examination of the disease and its impact on the country since the last report in 2018. This is a report from the scientific community to Crohn's and Colitis Canada, and the information and recommendations will inform perspectives, positions, and, hopefully, decision-making.

The Report this year will integrate the interpretation of the most recent literature through the eyes of patients and family partners.

Aim

This online survey aims to gather your perspectives and interpretation of the chapter that you received along with this survey link.

Procedure

- This survey will take you about 10 minutes to complete.
- The results will be reported in aggregate form and no names will be used.
- We kindly ask you to respond to all the questions in the survey.

- Please feel free to ask any questions regarding the procedures and goals of the survey or your role.

Potential Risks
There are no known or anticipated risks to you by participating in this work.

Potential Benefits

- You may receive no personal benefits from participation in this survey. The findings will enrich the interpretation of the scientific literature and inform the development of future reports.
- We offer you to be a co-author of the chapter of The Impact Report that you are revising and interpreting. If you agree, your name and affiliations will be displayed in publications, along with the information of the other co-authors.

Compensation
There is no compensation for participating in this survey.

Confidentiality

- Responses will be anonymized, and results will be reported in aggregate form.
- This survey is hosted by Survey Monkey. Your data will be stored in facilities hosted in Canada. Please see the following for more information on the SurveyMonkey privacy policy at https://www.surveymonkey.com/mp/legal/privacy/

Storage of Data

- Survey results, collected as electronic data, will be stored on a password-protected computer and backed up by Crohn's and Colitis Canada.
- Survey documents will not be accessible to individuals outside of the team preparing the Impact Report.

Right to Withdraw

- Participation in this survey is voluntary and you are free to choose to participate or not.
- You can decide not to participate at any time by closing your browser or choose not to answer any questions you do not feel comfortable with.
- Whether you choose to participate or not will have no effect on your relationship with Crohn's Colitis Canada or any of the team members of the Impact Report.

Follow up

- After completing this online survey and the group discussion, you will receive a copy of a paragraph summarizing the perspectives and interpretations made by patient and family partners of the assigned chapter. You will have the opportunity to provide your feedback on the drafted paragraph.
- The paragraph with your perspectives and interpretation will be integrated into the corresponding chapter of the Impact of IBD in Canada Report.
- You will receive the link to the scientific article and full report.

To obtain more information about this work, please contact Sara Ghandeharian (Manager, Patient Programs and Evaluation, Crohn's and Colitis Canada) at sghandeharian@crohnsandcolitis.ca

Please see below to provide your consent:

* 1. Do you provide your informed consent and understand the above conditions of participation in this survey?

◯ Yes (thank you for providing your consent to take part in this online survey)

◯ No (please contact sghandeharian@crohnsandcolitis.ca if you have questions)

Impact of IBD in Canada Report - Patient and Family Partner Feedback

Online Survey

Please answer the following questions to the best of your ability. We will invite you to provide additional feedback orally at a consensus meeting in 2023, after we incorporate your initial feedback from this survey.

2. What was the most relevant piece of information in the chapter to you?

3. Based on your response to Q2 (most relevant piece of information), what does this mean to you as a person living with IBD or a caregiver for someone living with IBD?

4. What do you think are the top three take-away messages from the chapter?

Take-away message 1

Take-away message 2

Take-away message 3

5. For Crohn's and Colitis Canada (CCC) to advocate to government or policy makers in the healthcare systems, they need to know what the most important messages from this chapter are.

What would you suggest are the most important parts of this chapter that CCC should advocate for on behalf of the IBD community?

Impact of IBD in Canada Report - Patient and Family Partner Feedback

Glossary of Terms Feedback

6. Thinking about the Glossary, do the definitions of the terms convey enough information?

◯ Yes

◯ No

7. Do you feel like you gained an understanding of the defined medical terms?

◯ Yes

◯ No

8. If you answered "No" to Q6 or Q7, which terms were difficult to understand or did not convey enough information?

9. Were there any terms from the chapter you read you would like added to the glossary?

Impact of IBD in Canada Report - Patient and Family Partner Feedback

10. Please provide a quote about the research that will be published along with the public release of the report.

11. What is the greatest strength of the chapter?

12. What is the greatest weakness of the chapter?

13. Are there any additional topics you think should be covered in this chapter?

14. If you have any other comments about the chapter, please write them here:
